# Supplementary material for: Contrasting genomic epidemiology between sympatric Plasmodium falciparum and Plasmodium vivax populations
Source: Nat Commun. 2024 Sep 30;15:8450. doi: 10.1038/s41467-024-52545-6 (PMC11442626; doi:10.1038/s41467-024-52545-6)
Supplement: Supplementary file 1 — Supplementary Information [file 41467_2024_52545_MOESM1_ESM.pdf]

Supplementary Information

Contrasting genomic epidemiology between sympatric *Plasmodium falciparum* and *Plasmodium vivax* populations

Philipp Schwabl, Flavia Camponovo, Collette Clementson, Angela M. Early, Margaret Laws, David A. Forero-Peña, Oscar Noya, María Eugenia Grillet, Mathieu Vanhove, Frank Anthony, Kashana James, Narine Singh, Horace Cox, Reza Niles-Robin, Caroline O. Buckee, Daniel E. Neafsey

This file includes:

Supplementary Figures 1-23

Supplementary Table 1

Supplementary Texts 1-2

Supplementary References

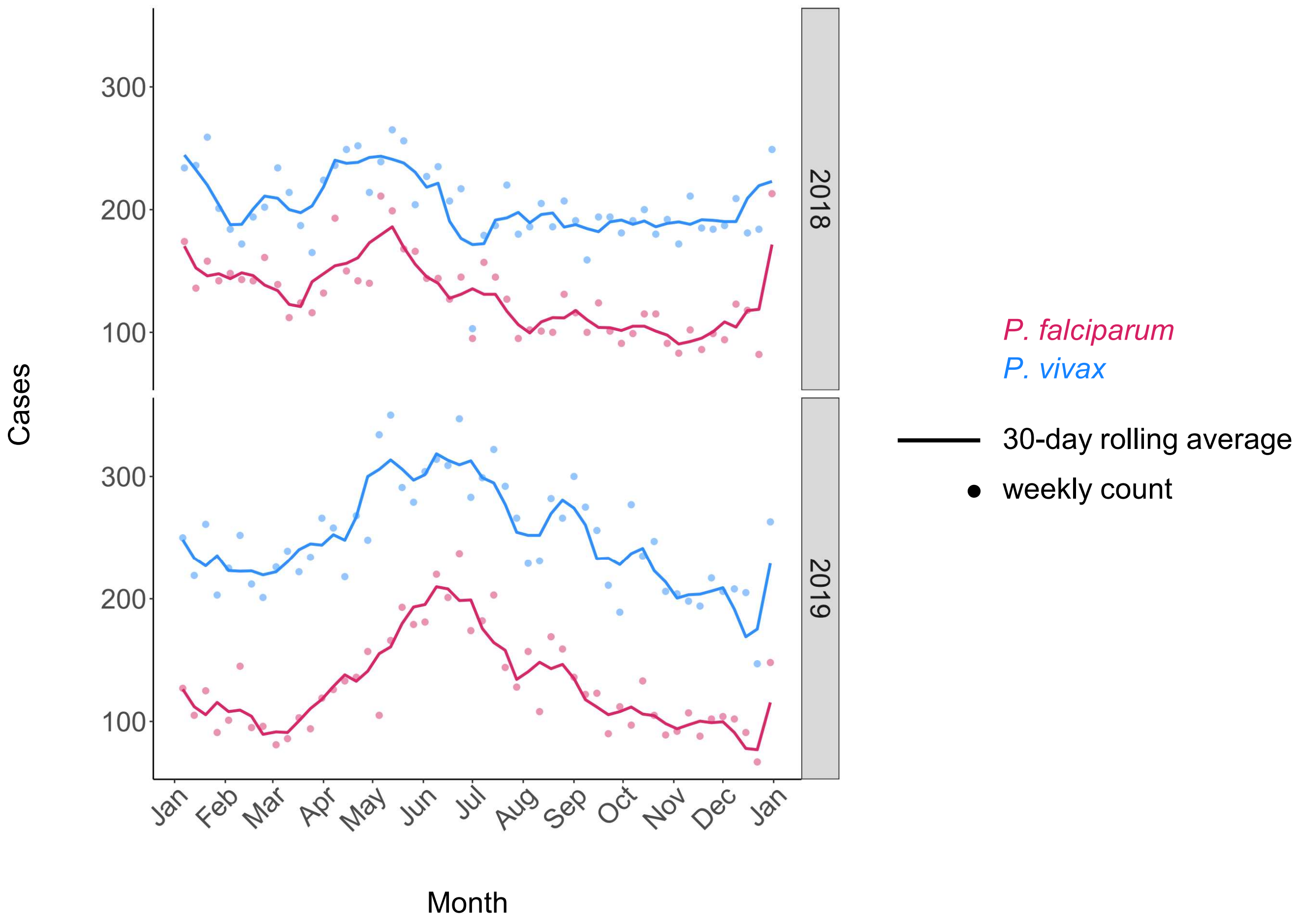

Supplementary Fig. 1 Intra-annual variation in *P. falciparum* and *P. vivax* cases in Guyana. Points represent weekly reported cases and lines represent 30-day rolling averages in 2018 and 2019.

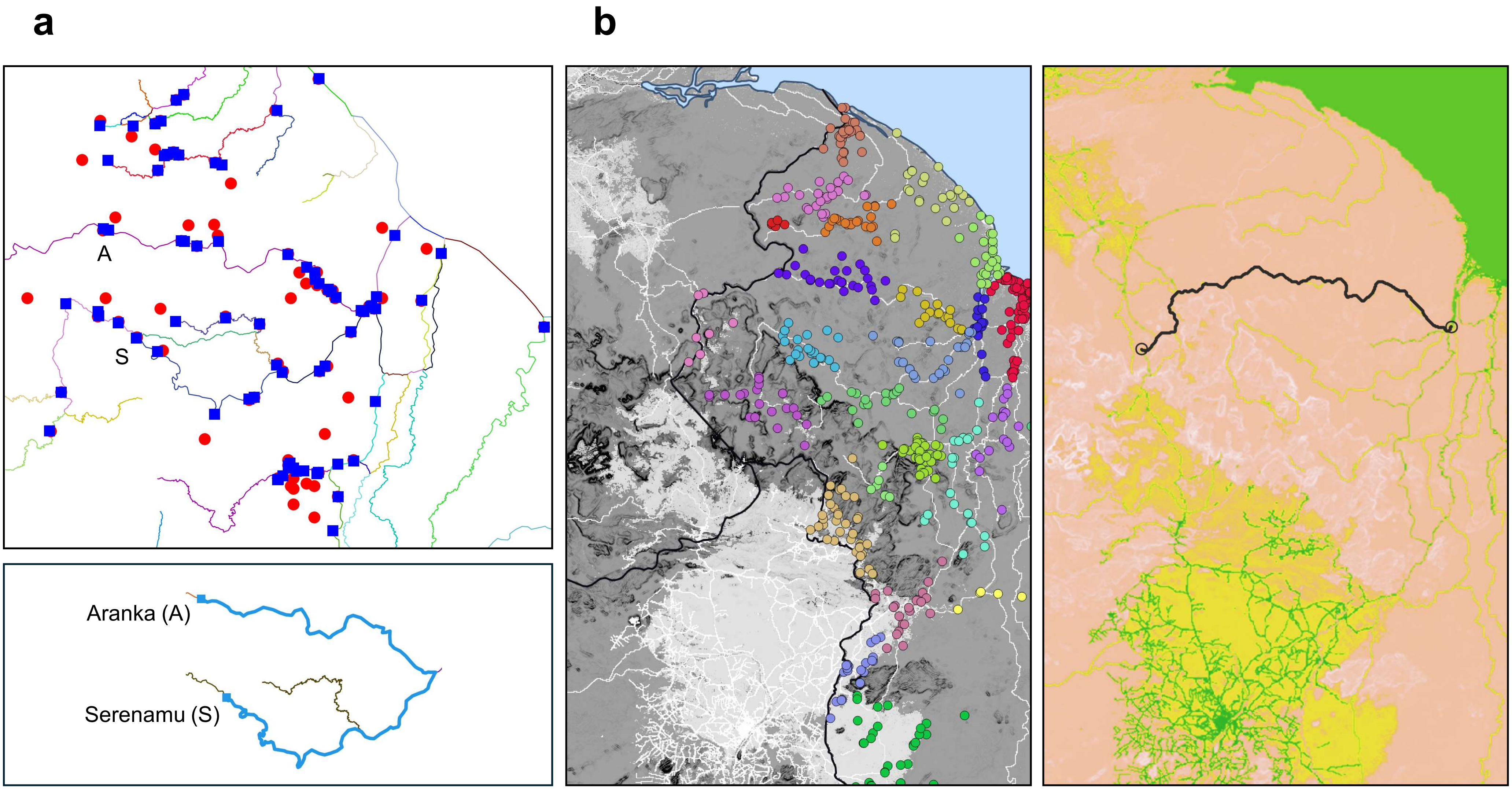

Supplementary Fig. 2 Epidemiological zoning based on connectivity analysis in Guyana. Diagnosis and infection localities (inferred based on reported stay 2 weeks prior to diagnosis) were grouped into 27 epidemiological zones. Though not automated using any fixed thresholds, grouping decisions were partially informed by river distance calculation and resistance surface analysis. **a)** To measure river distances, localities were first fitted to nearest river coordinates (red and blue points, respectively). Distances along rivers were then measured between fitted coordinates (e.g., Aranka to Serenamu, bottom plot). These analyses used the RIVERDIST[1] package in R. River/road lines in the map represent coordinate series collected by browsing OpenStreetMap data (Open Database License by Open Data Commons; http://opendatacommons.org/licenses/odbl/1.0/). **b)** For resistance surface analysis, localities were plotted on a mobility friction map obtained from https://malariaatlas.org/project-resources/accessibility-to-healthcare/ representing work by Weiss et al. 2018[2]. Light grey and white represent highest conductance to motorized travel. The right plot illustrates how a least cost path (black line inferred via GDISTANCE[3] package in R) for Las Cristinas (Venezuela) to Bartica (Guyana)) matches the flow of the Cuyuni River. Yellow and green represent highest conductance to motorized travel.

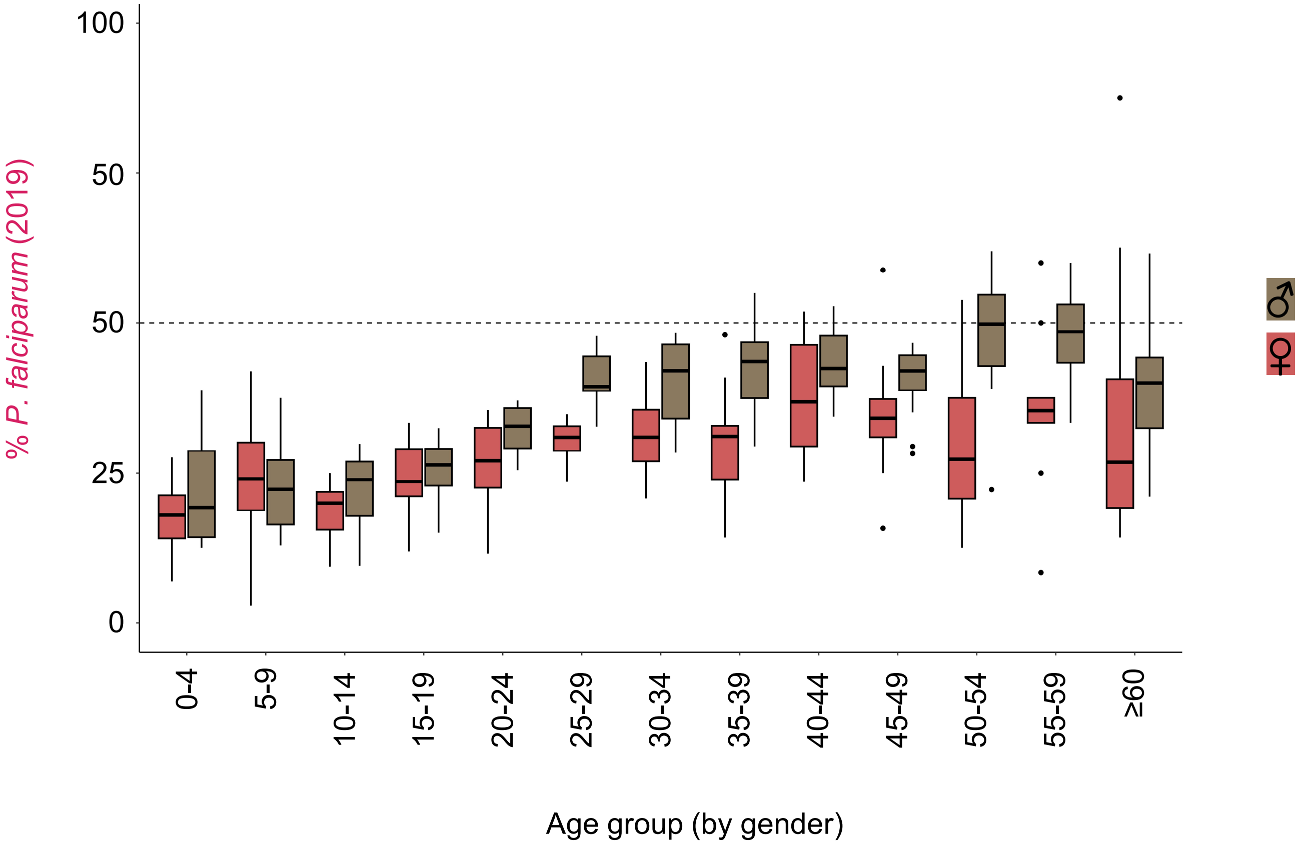

Supplementary Fig. 3 Relative *P. falciparum* prevalence with respect to age and gender in Guyana. Boxplots summarize monthly variation (median and quartiles) in the percent of malaria cases representing *P. falciparum* in 2019. Brown and red represent values for male and female cases, respectively.

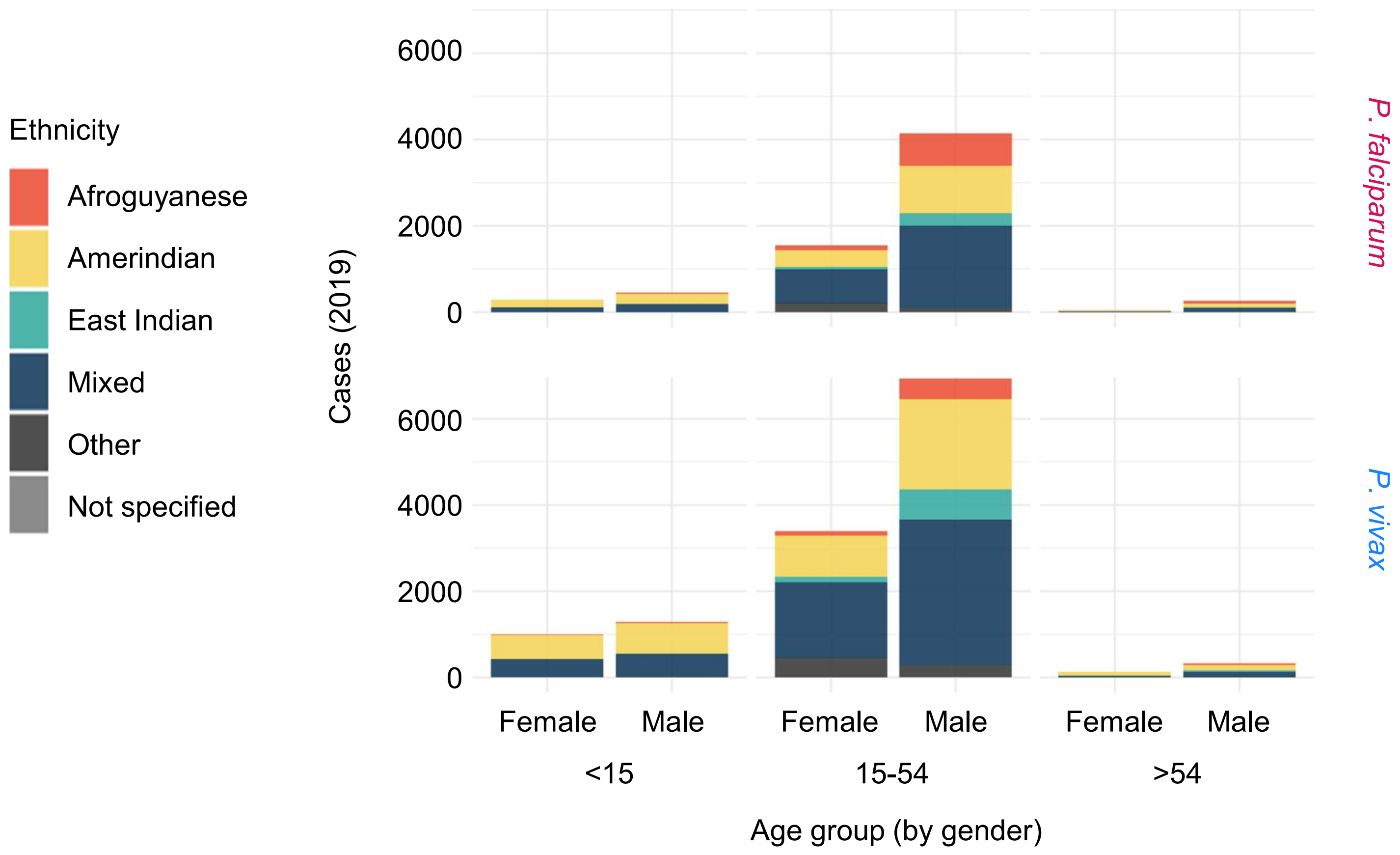

Supplementary Fig. 4 *P. falciparum* and *P. vivax* case counts be age, gender, and ethnicity in Guyana. Colors in stacked bar charts indicate self-reported ethnicities (key at left) among malaria patients in 2019.

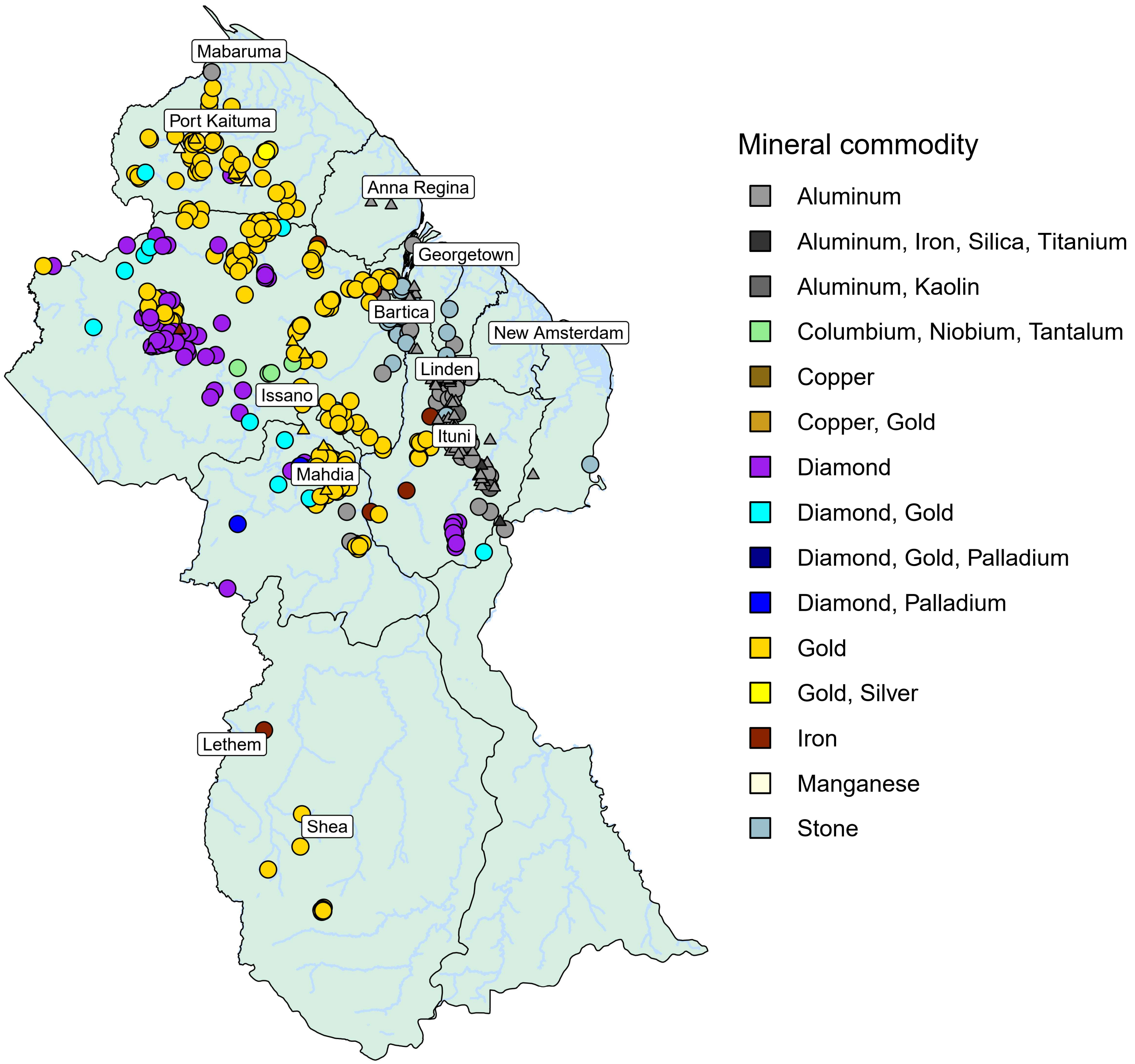

Supplementary Fig. 5 Metallic and non-metallic mineral deposits in Guyana. The map plots mining deposit coordinates (points) and corresponding commodities (point colors) recorded by the US Geological Survey (USGS) Mineral Resources Data System (MRDS). Selected cities/townships are also shown (labels centered on coordinates). The MRDS (available at https://mrdata.usgs.gov/mrds/) indicates one or more primary commodities (i.e., commodities which are recovered and have a strong effect on the economic viability of the site) for 473 of 583 (81.1%) sites listed for Guyana. These 473 sites are plotted as circles. Additional information on secondary commodities (i.e., commodities which can be economically recovered but have little effect on the economic viability of the site) is not included on the map. The MRDS indicates only tertiary commodities (i.e., commodities which are economically interesting but not considered recoverable at the time of analysis) for 110 of 583 (18.9%) sites. These 110 sites are plotted as smaller triangles and are colored based on tertiary commodities. Commodities within observed sets (see legend) are ordered alphabetically. Please note that the MRDS represents a collation of various individual commodity reports, and in the case of Guyana, these are primarily geological surveys from the mid 1900s. The map is therefore not a direct reflection of contemporary exploitation or exploration patterns but an indicator of underlying geological characteristics. Blue lines represent OpenStreetMap waterway data distributed under the Open Database License by Open Data Commons (http://opendatacommons.org/licenses/odbl/1.0/).

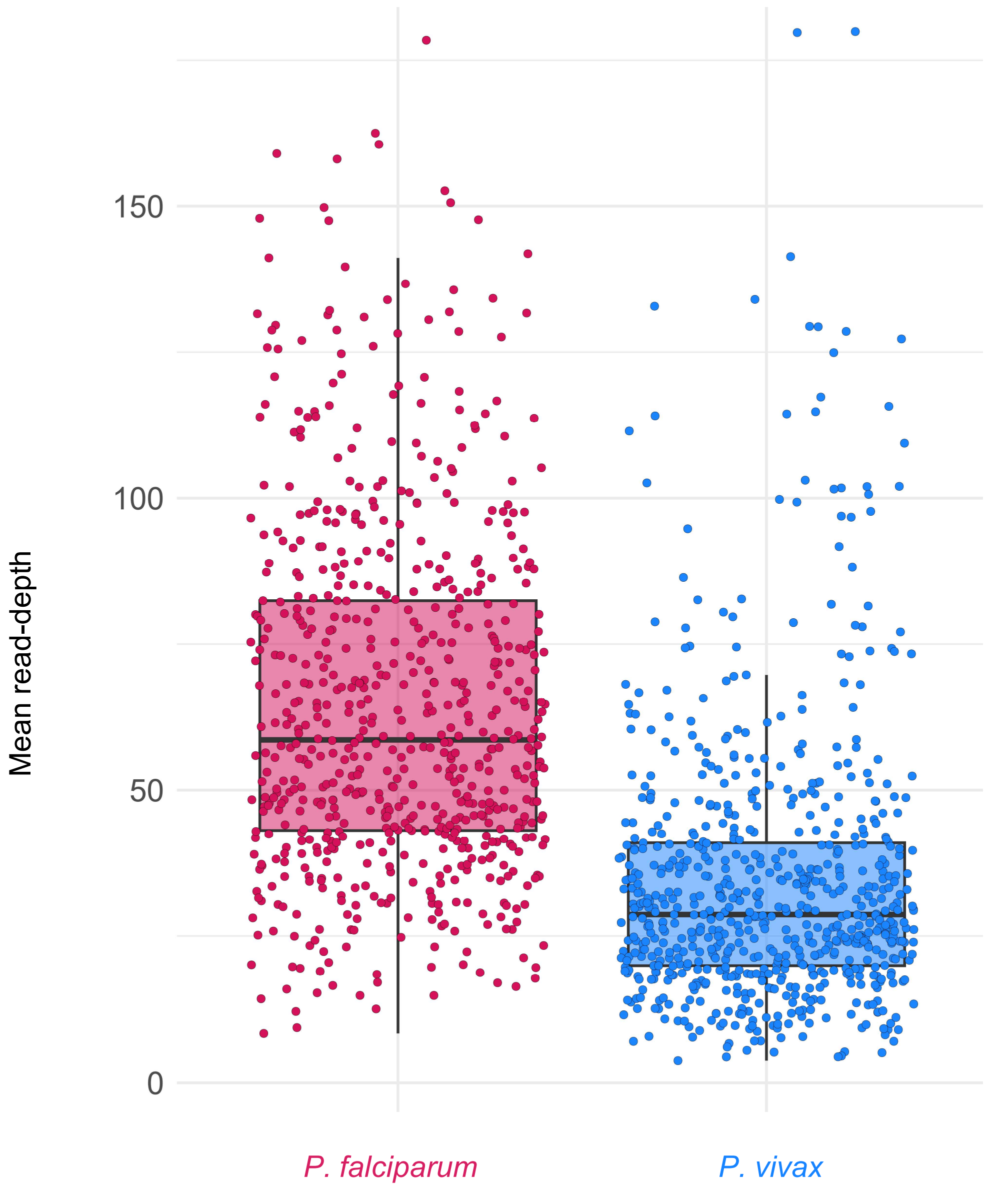

Supplementary Fig. 6 Sequencing depth of *P. falciparum* and *P. vivax* samples analyzed in the study. Boxplots summarize variation (median and quartiles) in mean read-depth associated with each sample (points). One outlier *P. falciparum* sample (G7B667, mean read-depth = 262.9) is not shown.

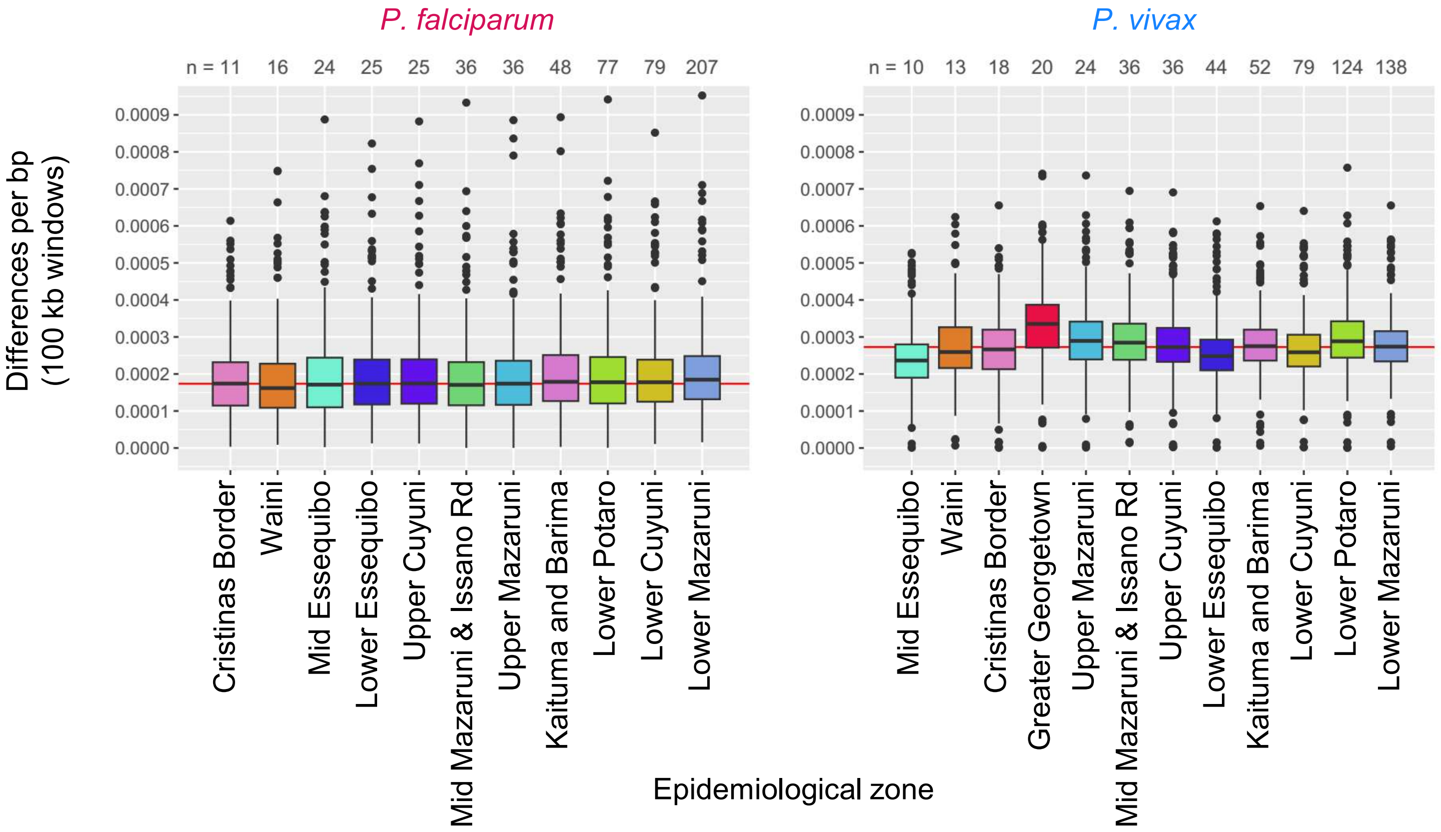

Supplementary Fig. 7 *P. falciparum* and *P. vivax* pairwise nucleotide diversity among epidemiological zones in Guyana. Boxplots summarize variation (median and quartiles) in windowed π values (y-axis) for each epidemiological zone (x-axis) represented by ≥10 genomic samples (see top) in 2020-21.

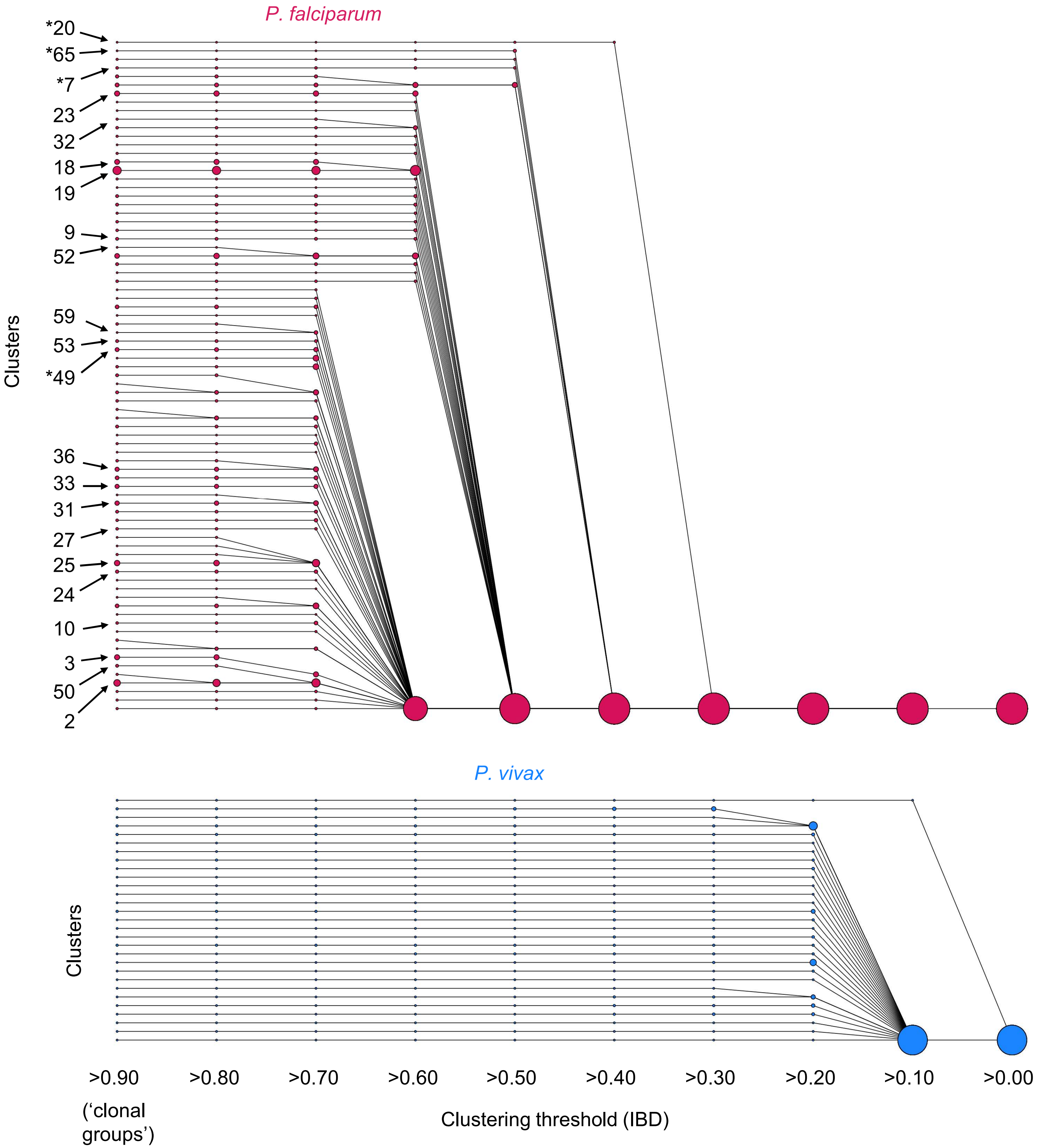

Supplementary Fig. 8 ***P. falciparum*** **and** ***P. vivax*** **clustering relationships in Guyana and Venezuela.** Clusters (circles) represent groups of samples in which each sample is related to at least one other sample in the group at the specified IBD threshold (x-axis, decreasing from left). Circle sizes represent cluster membership sizes, minimally two. Clusters adjacent on the x-axis are connected by a line if the right (lower threshold) cluster contains all the members of the left (higher threshold) cluster. Arrows at left indicate *P. falciparum* clonal group IDs mapped in Supplementary Fig. 10. Analysis includes samples from Guyana (2020-21) and Venezuela (2015-16 and 2019).

a

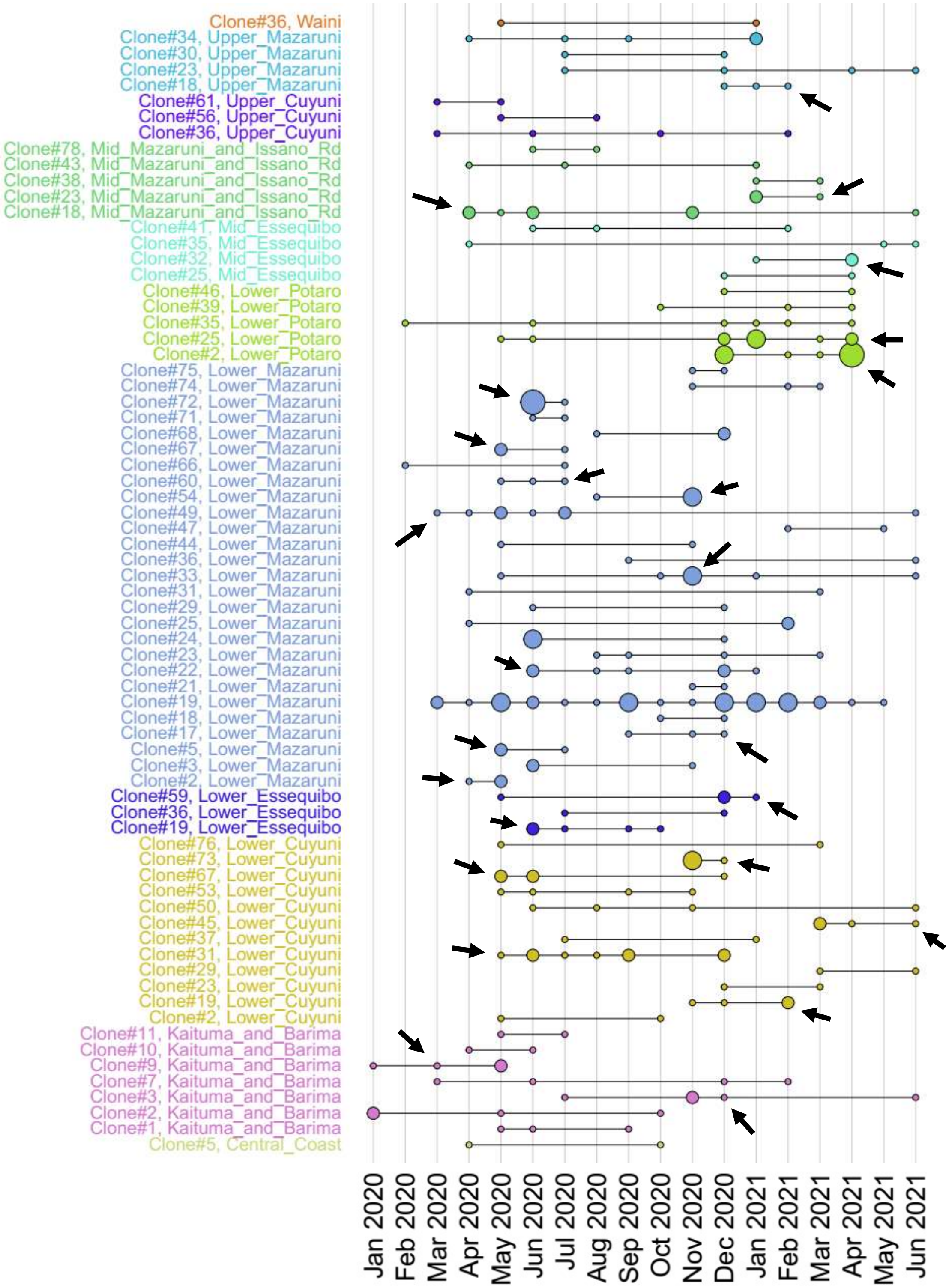

(Supp. Fig. 9 – continues on next page)

b

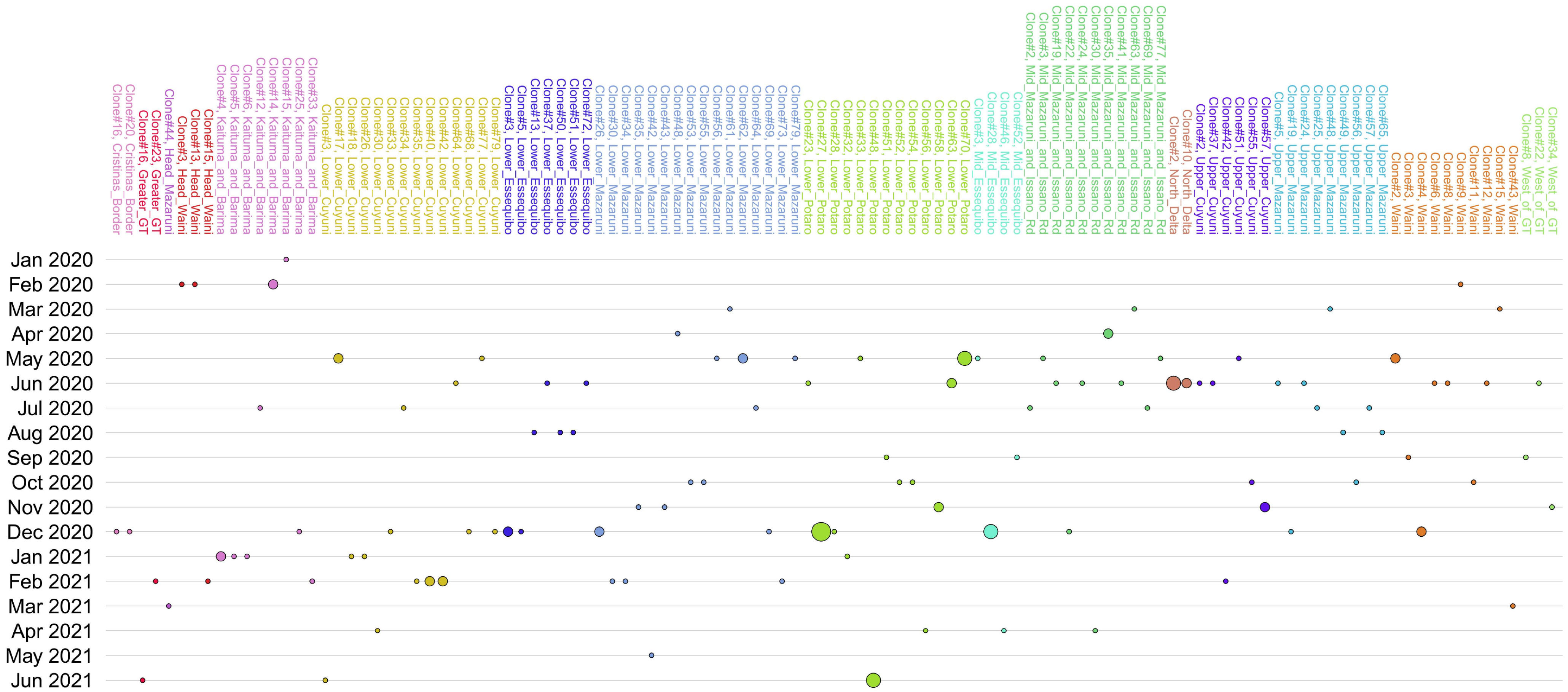

Supplementary Fig. 9 Spatiotemporal variation in *P. falciparum* clonal group membership in Guyana. The y-axis lists the clonal groups detected in each epidemiological zone (colors) in 2020-21. Group detection (circle size represents the number of group members detected) is plotted by month on the x-axis. **a)** When detections occur in multiple months per zone, these are connected by black lines. Arrows indicate instances where detection is temporally aggregated, i.e., a 4 month window contains ≥3 group members and also represents >50% of all members within that zone. **b)** In the majority of zones, membership detection does not occur in multiple months. Plots are split into two pages using flipped axes only to fit figure dimensions.

a

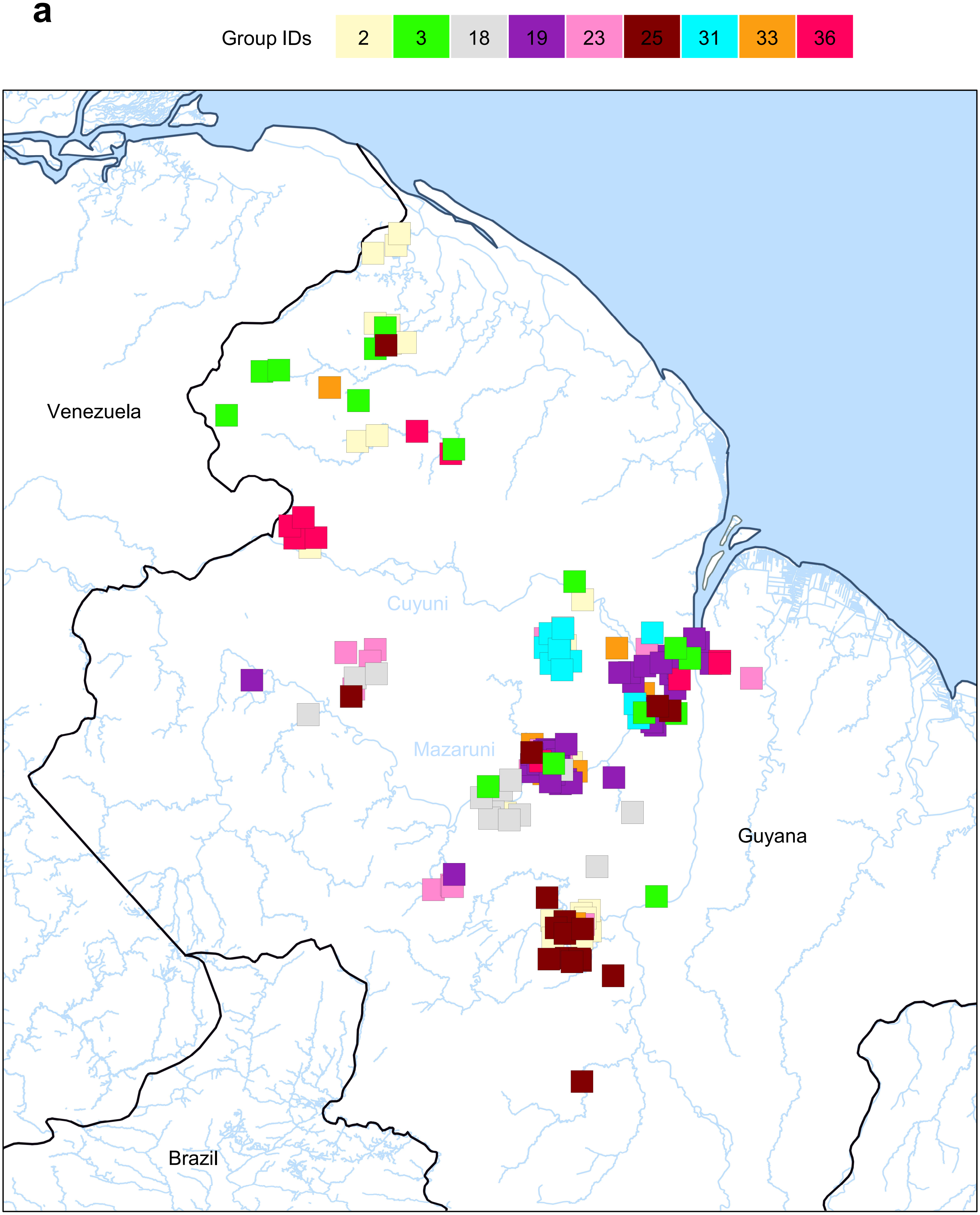

(Supp. Fig. 10 – continues on next page)

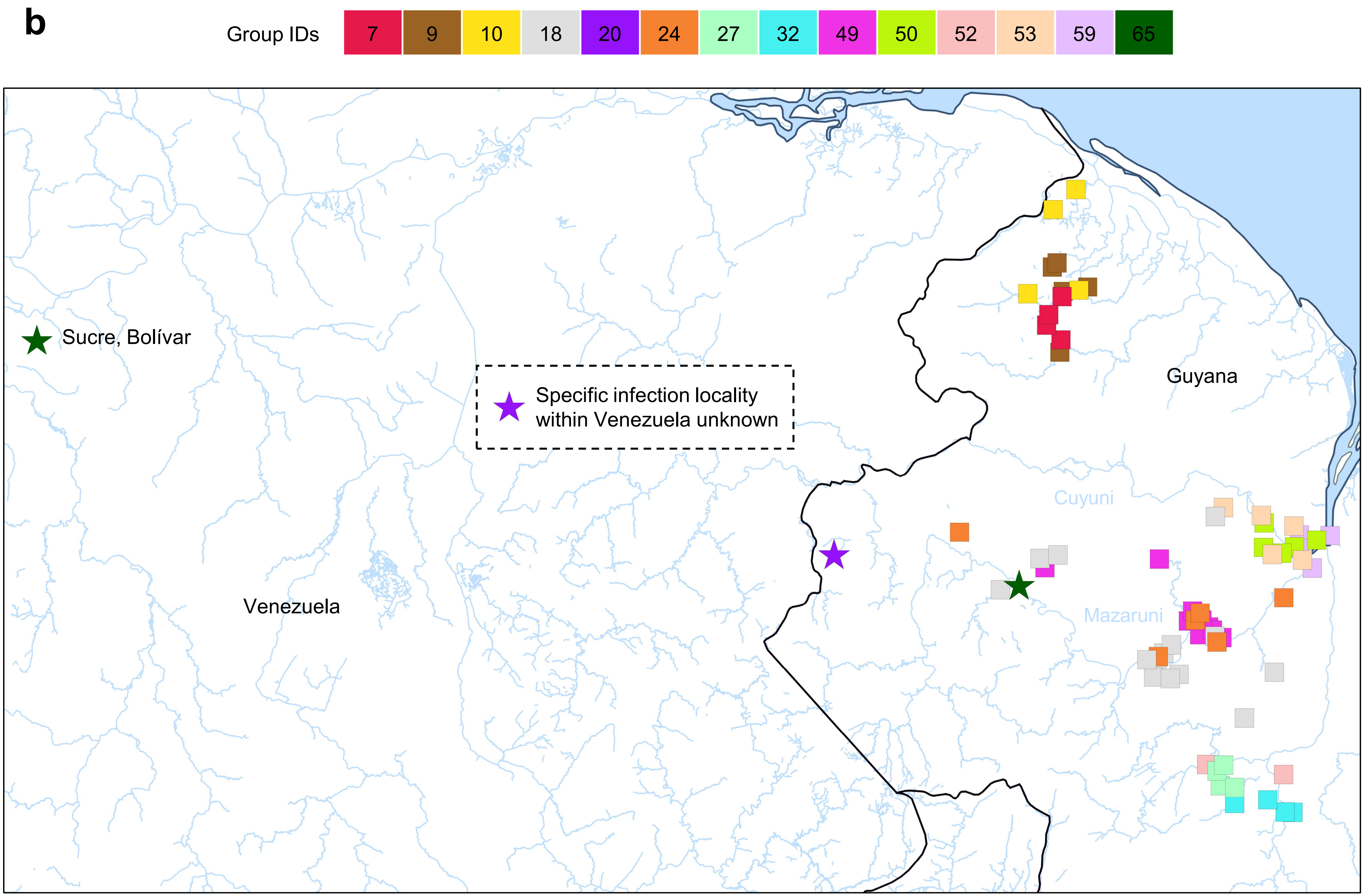

Supplementary Fig. 10 *P. falciparum* clonal group mapping in Guyana and Venezuela. a) Large clonal groups (≥10 members each) in 2020-21. **b)** Selected clonal groups highlighted in the main text (2015, 2019, and 2020-21). Colors indicate group IDs and star symbols indicate groups with partial membership in Venezuela (#65 in Sucre municipality (2019), Bolívar state, and #20, specific locality unknown (2015)). Slight jitter (±0.1 degrees) is applied to reduce overlap. Blue lines represent OpenStreetMap waterway data distributed under the Open Database License by Open Data Commons (http://opendatacommons.org/licenses/odbl/1.0/).

a

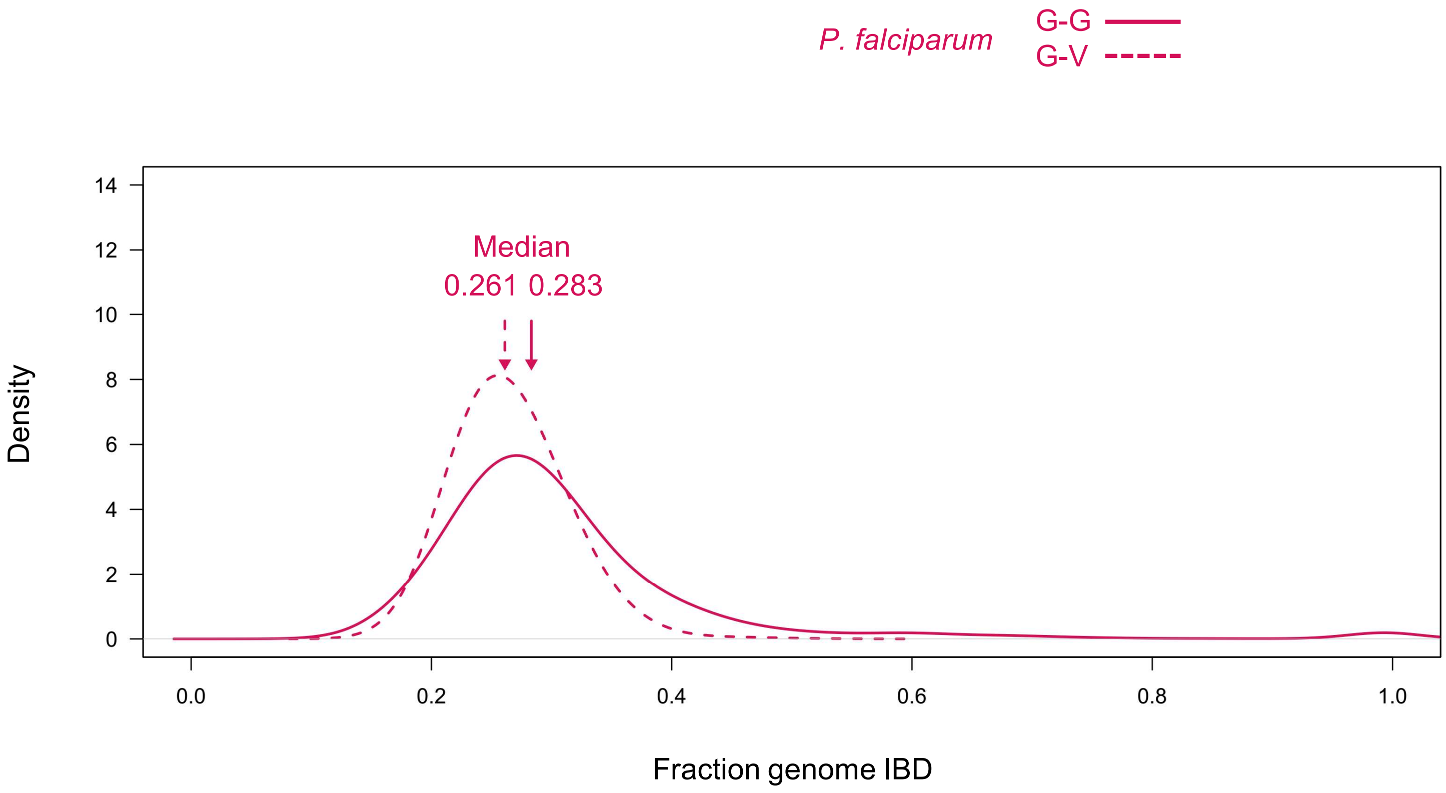

(Supp. Fig. 11 – continues on next pages)

b

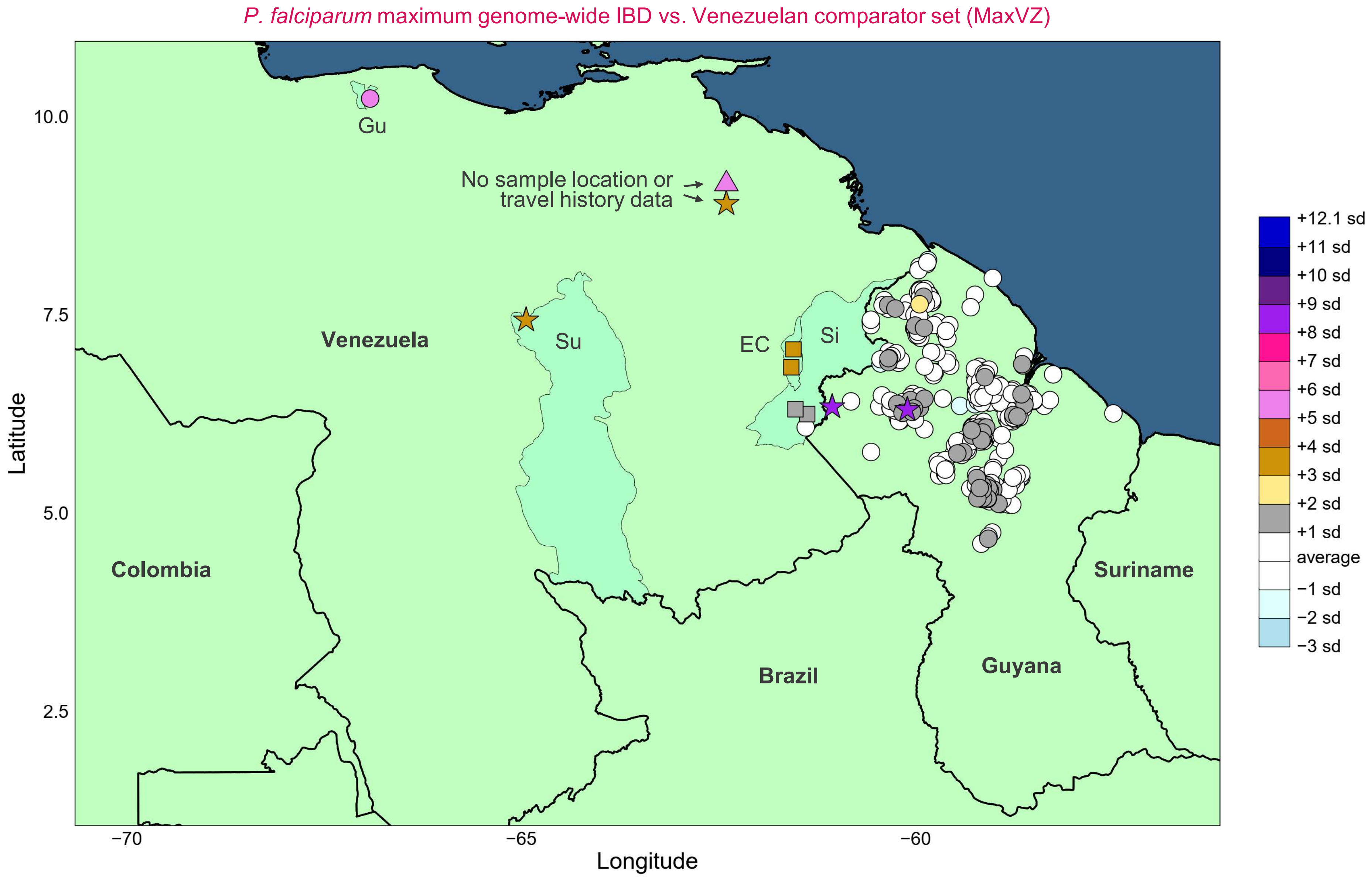

(Supp. Fig. 11 – continues on next pages)

c

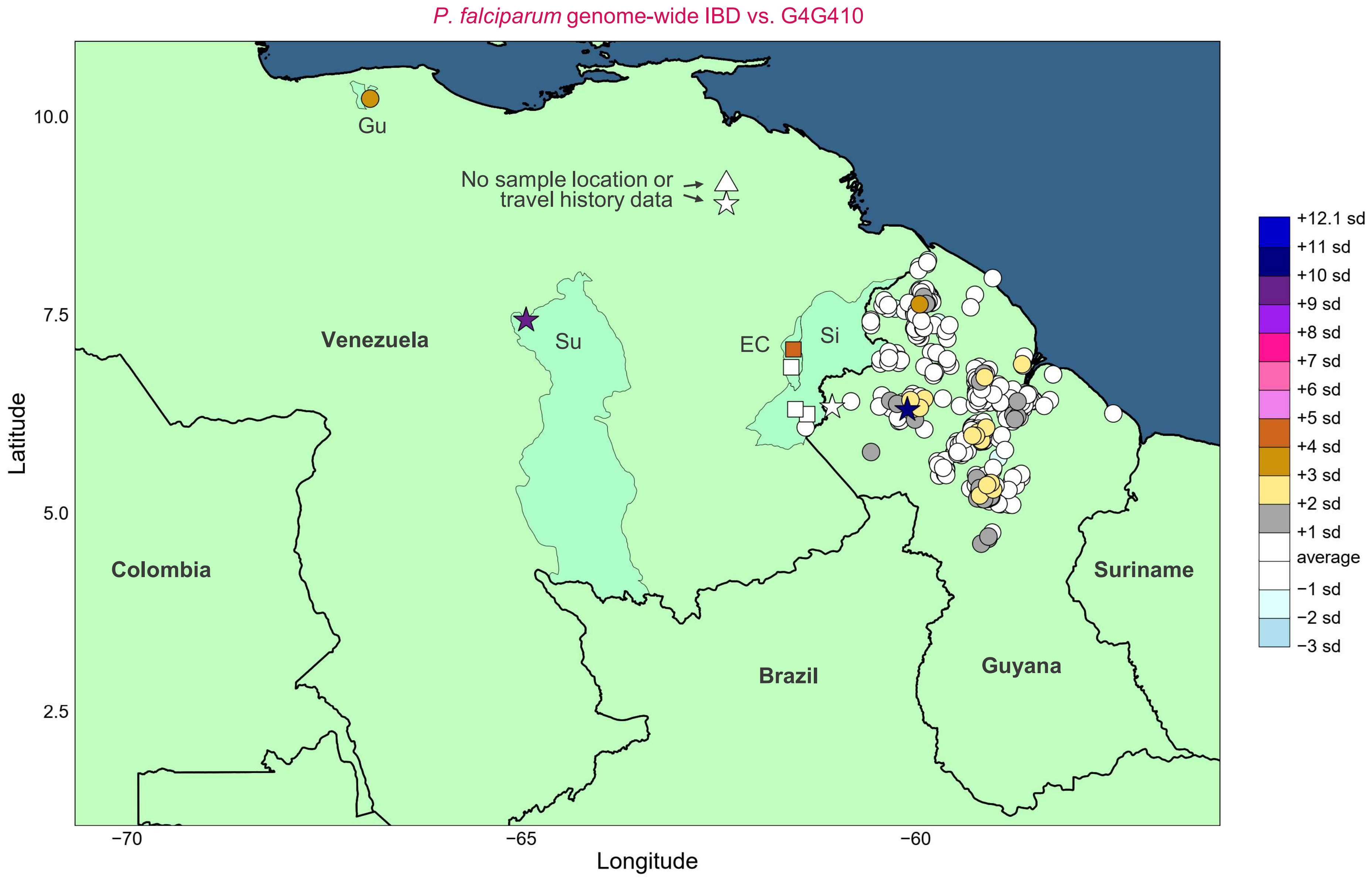

(Supp. Fig. 11 – continues on next pages)

d

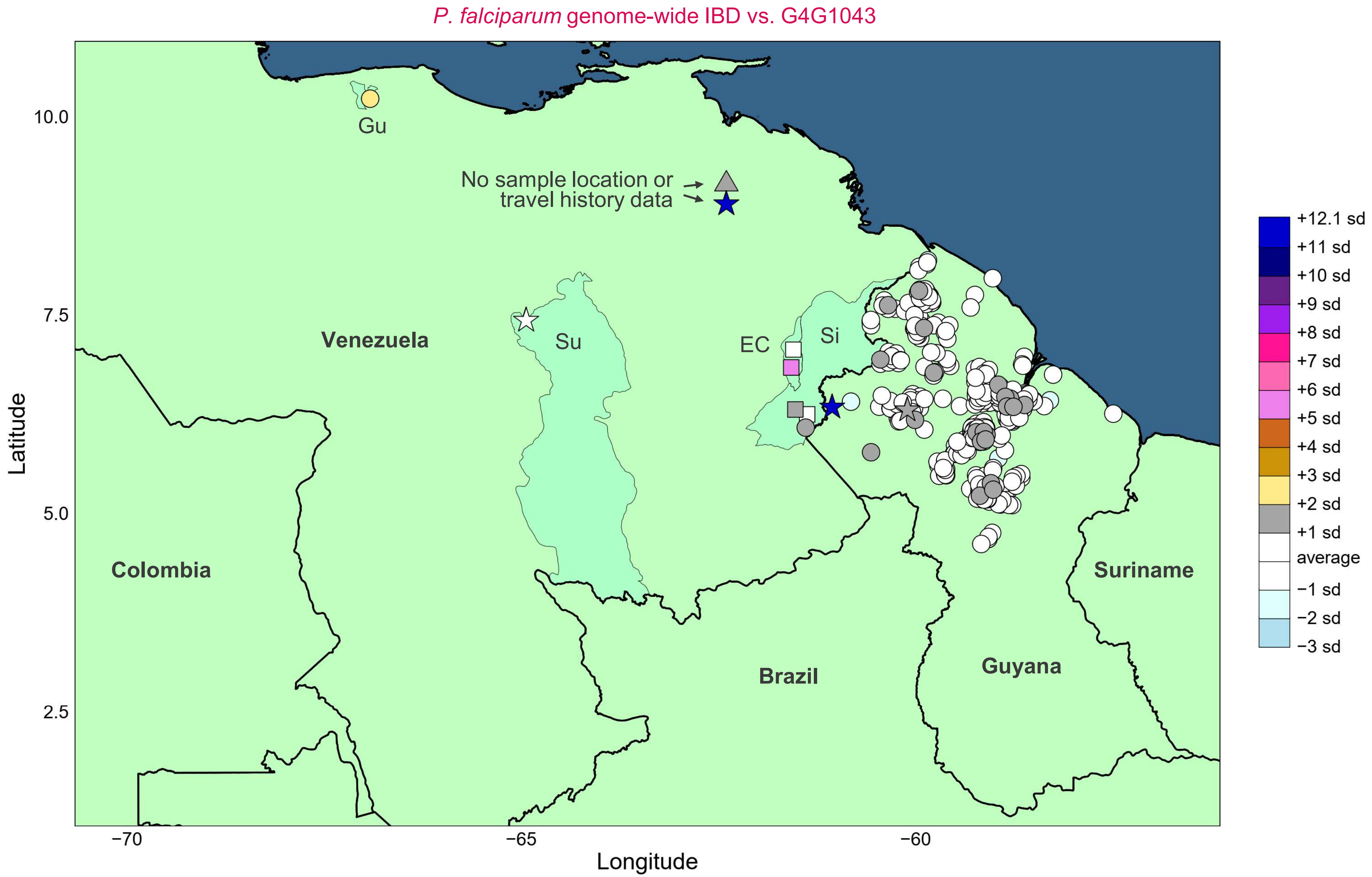

(Supp. Fig. 11 – continues on next pages)

e

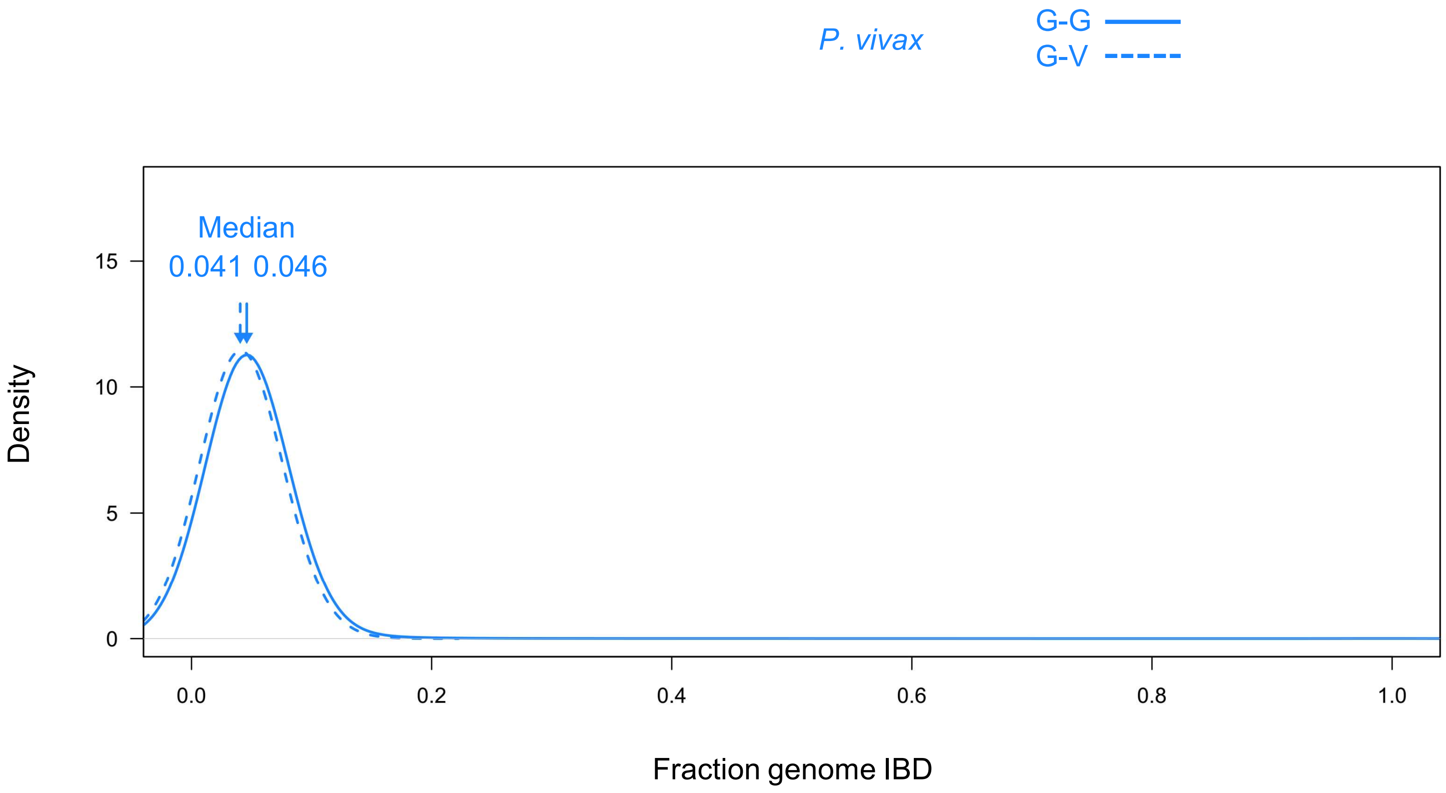

(Supp. Fig. 11 – continues on next pages)

f

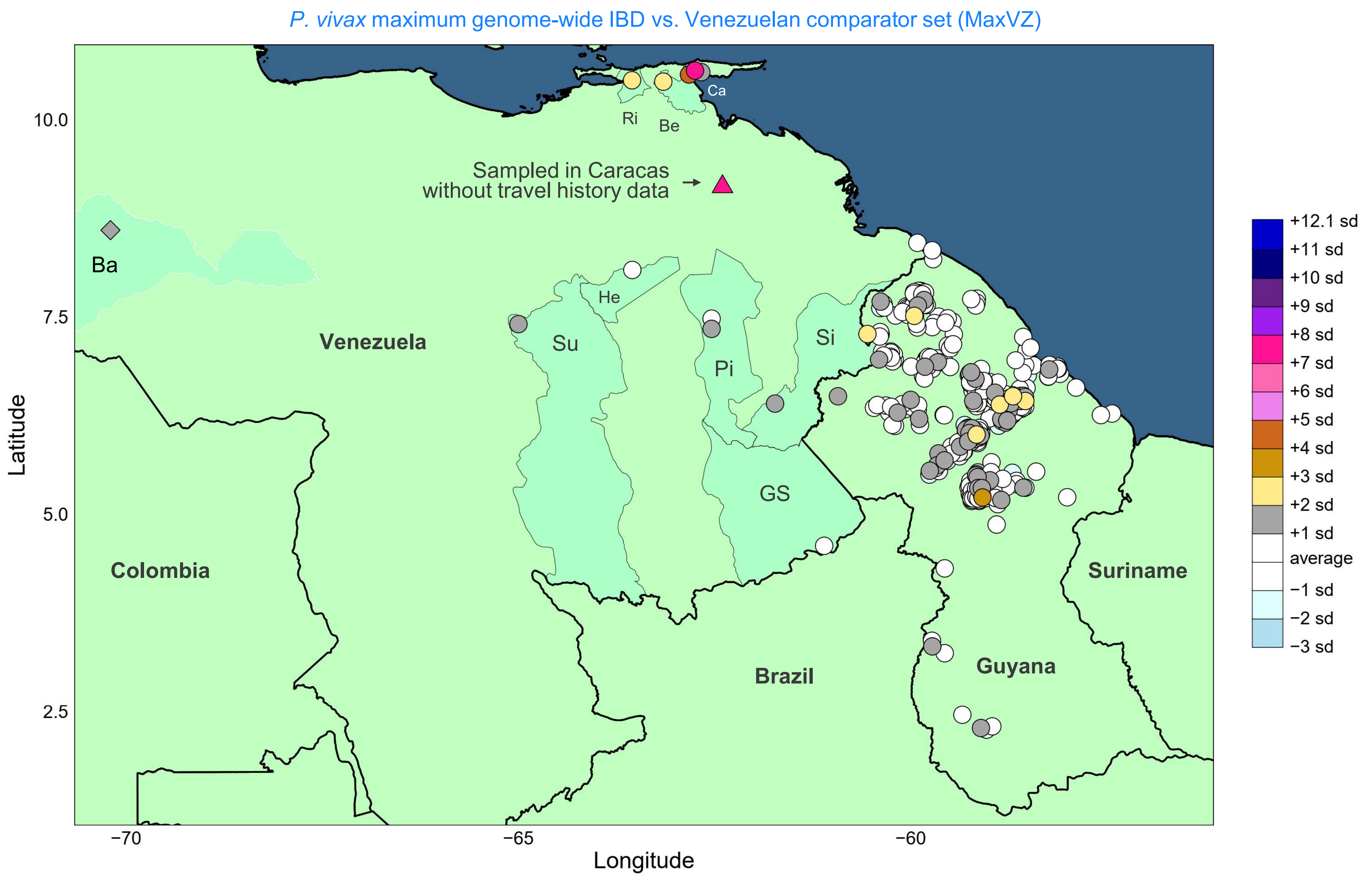

(Supp. Fig. 11 – continues on next pages)

g

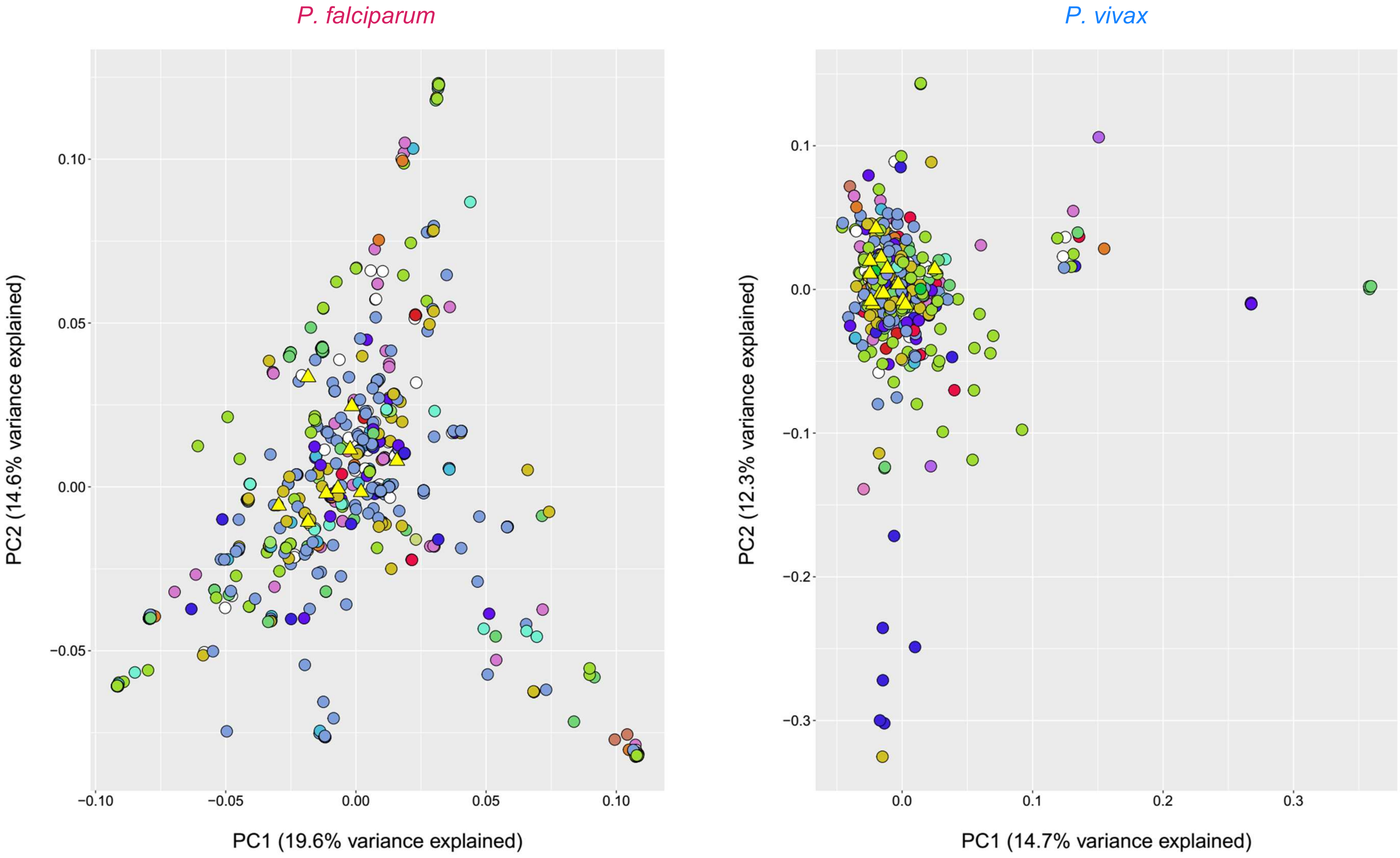

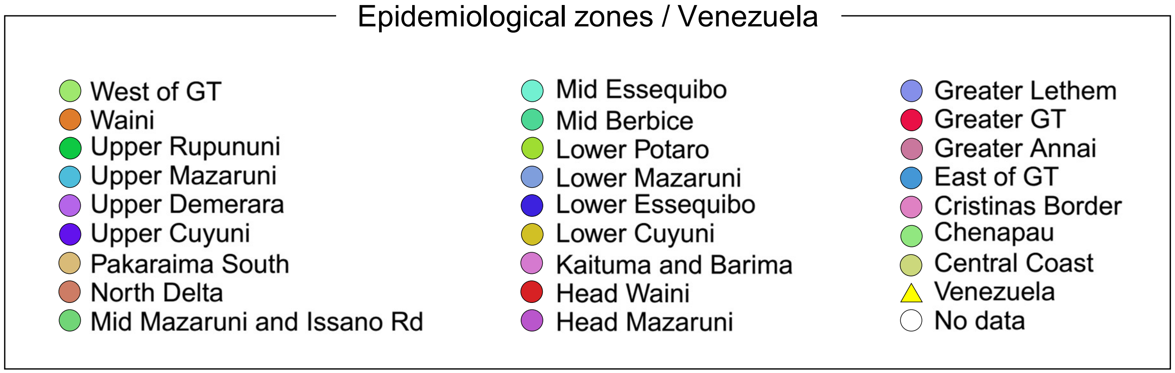

(Supp. Fig. 11 – continues on next pages)

h

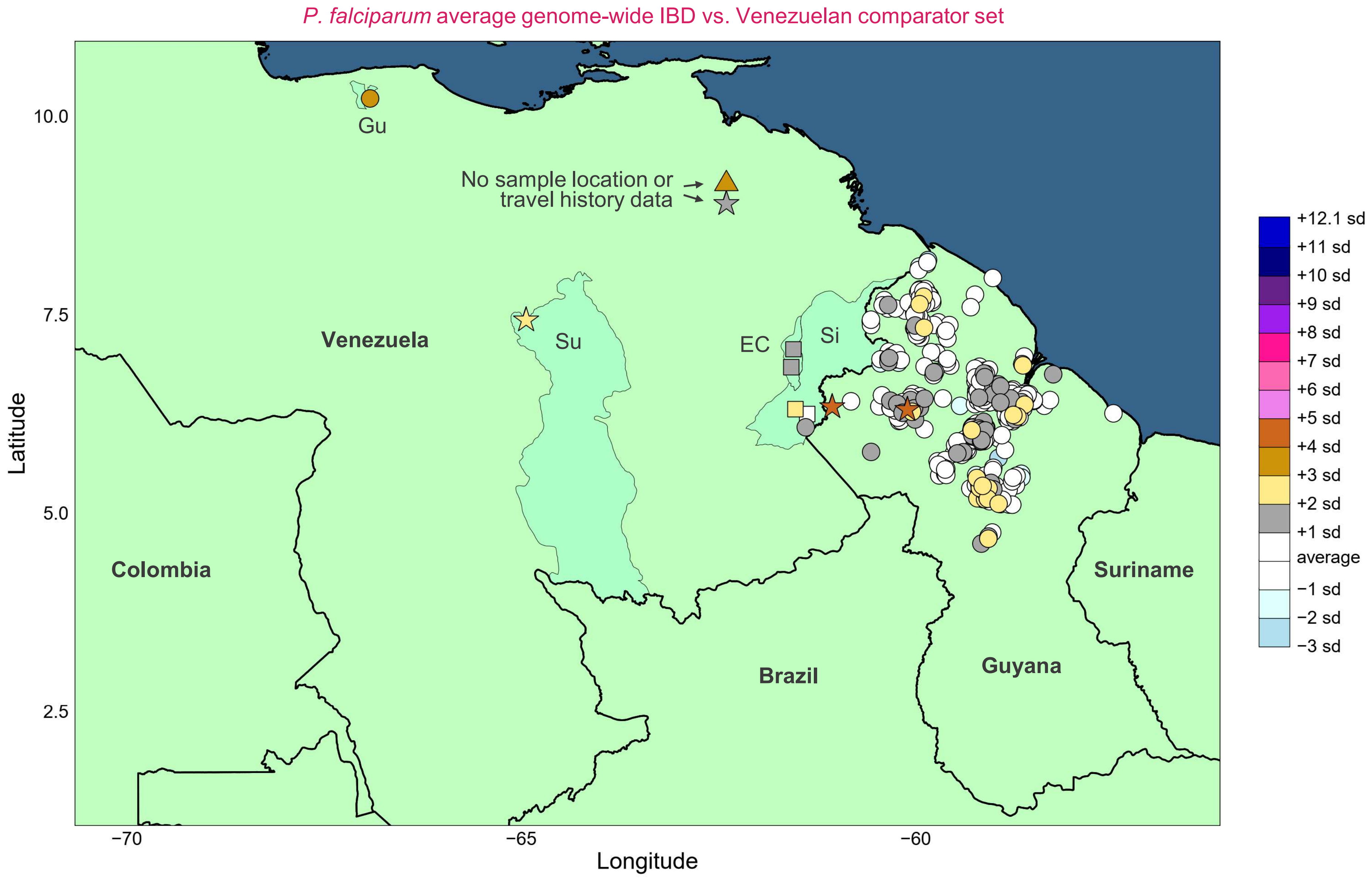

(Supp. Fig. 11 – continues on next pages)

i

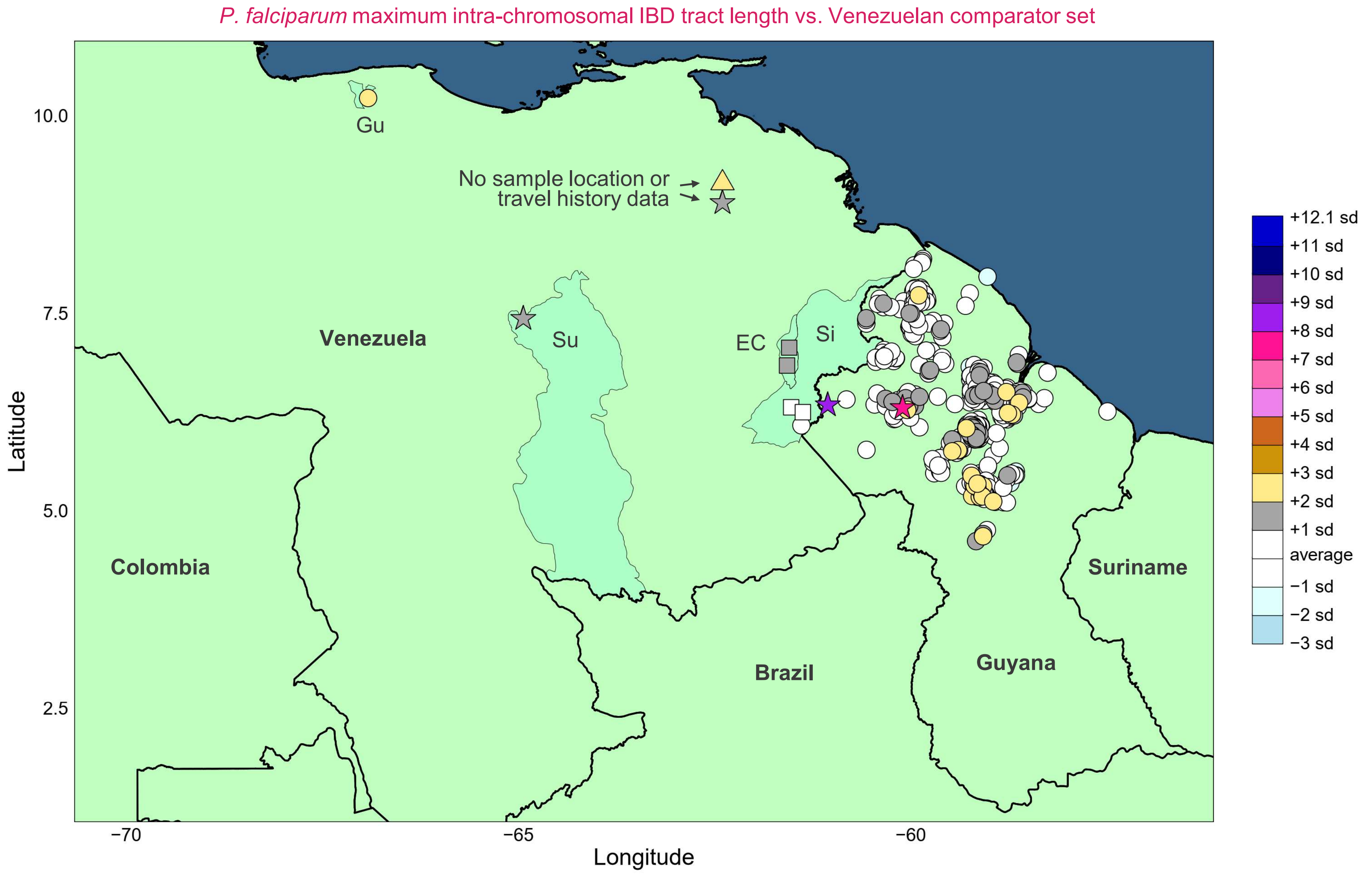

(Supp. Fig. 11 – continues on next pages)

j

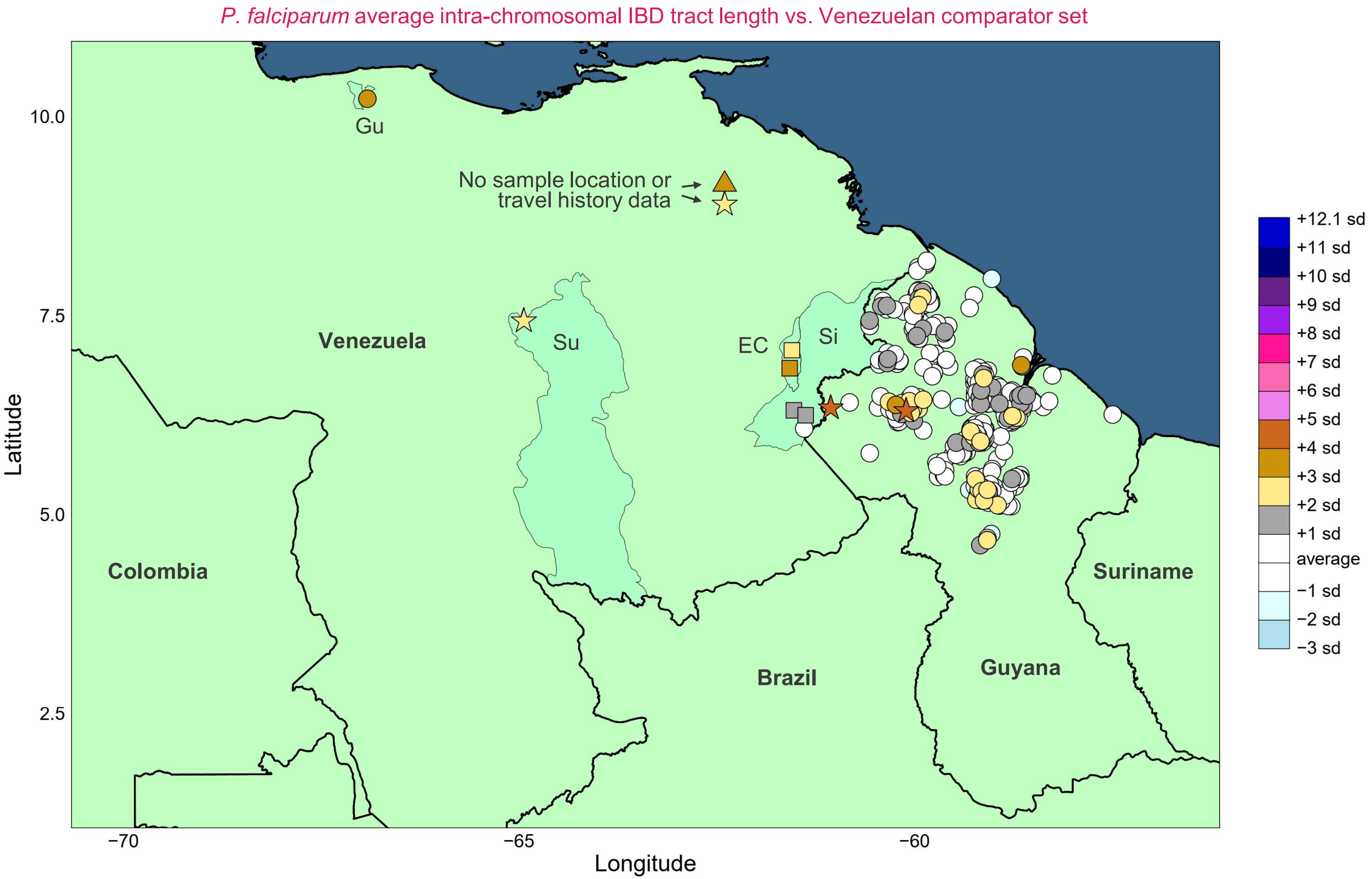

(Supp. Fig. 11 – continues on next pages)

k

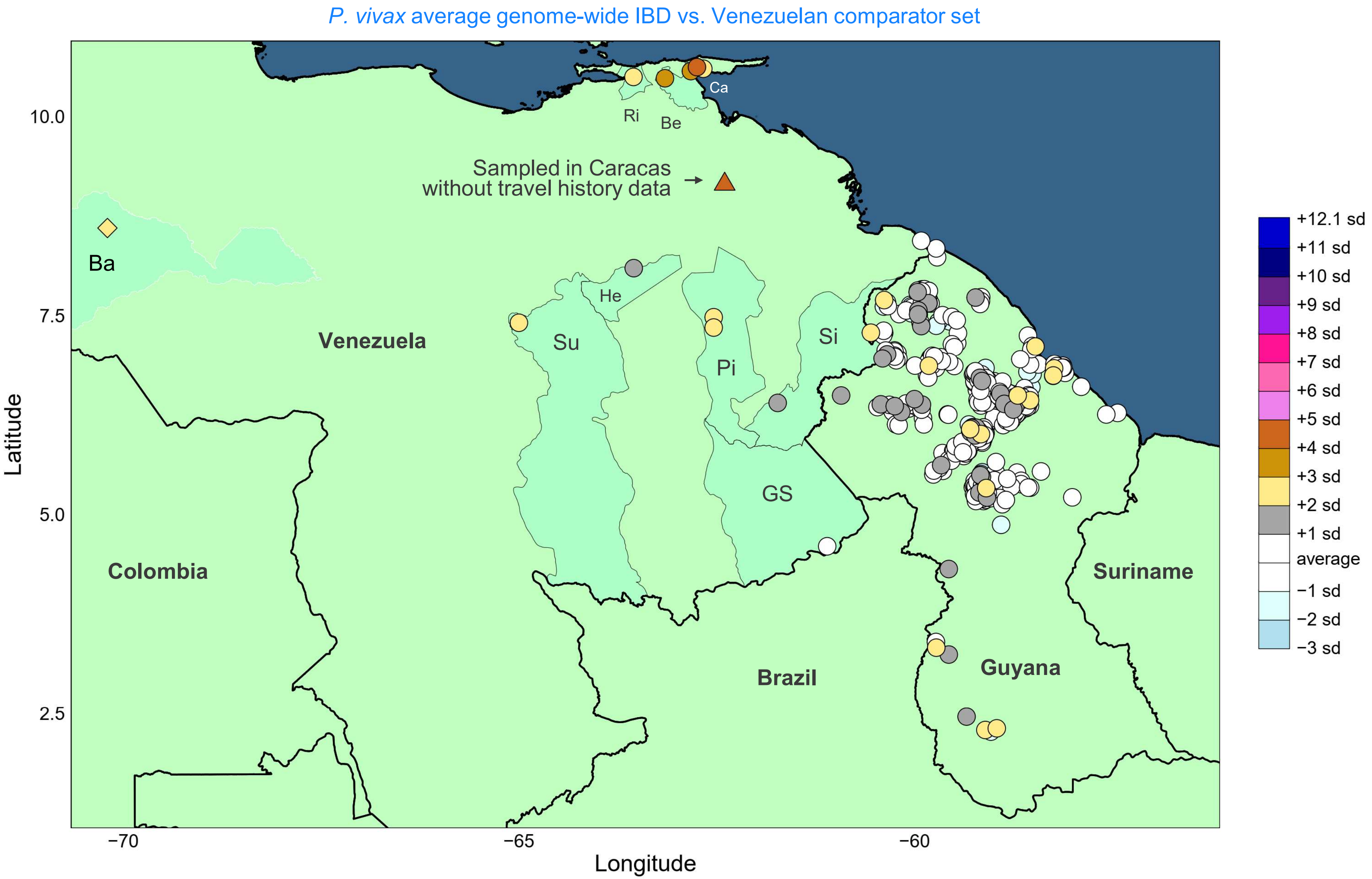

(Supp. Fig. 11 – continues on next pages)

I

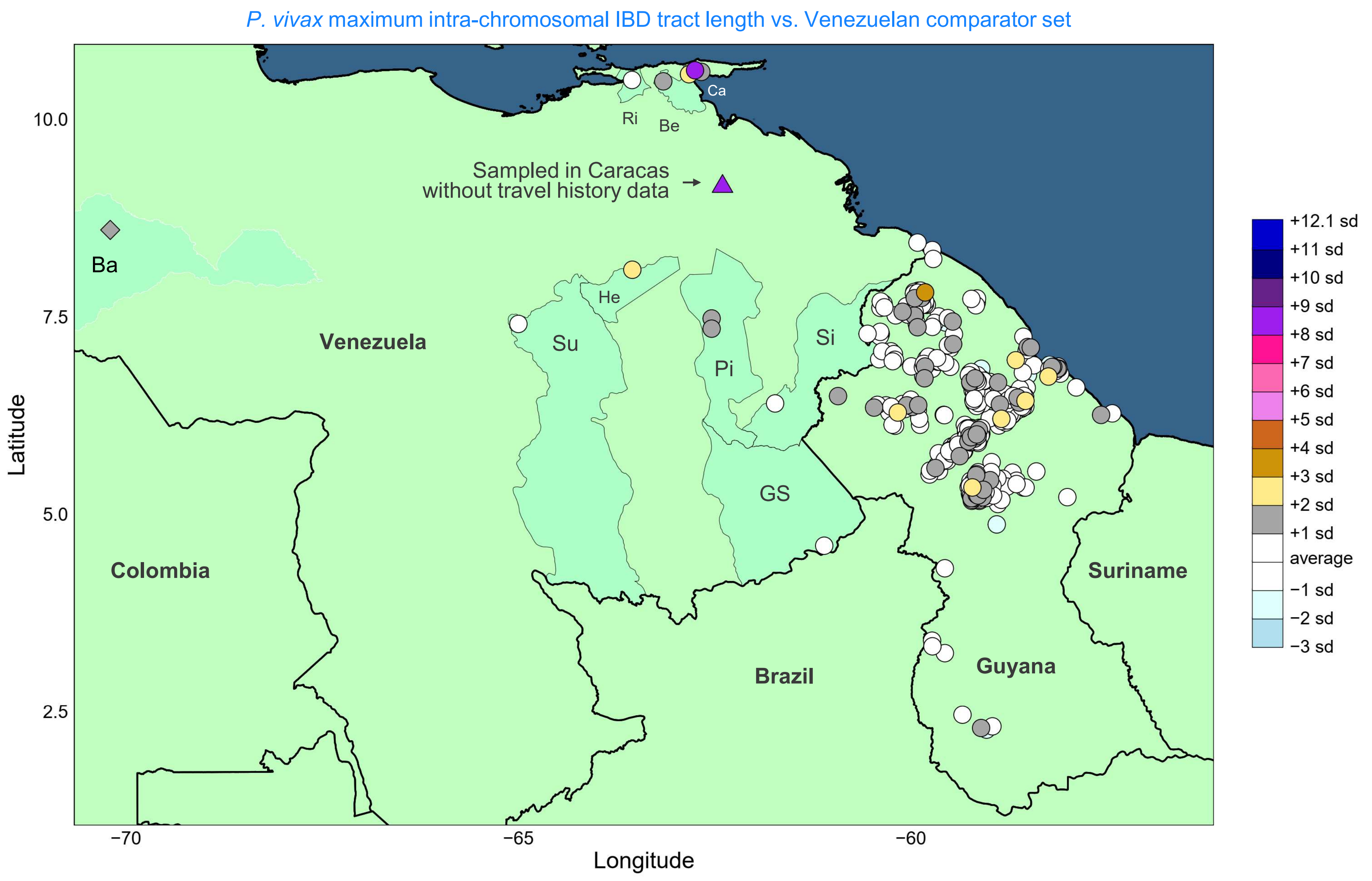

(Supp. Fig. 11 – continues on next pages)

m

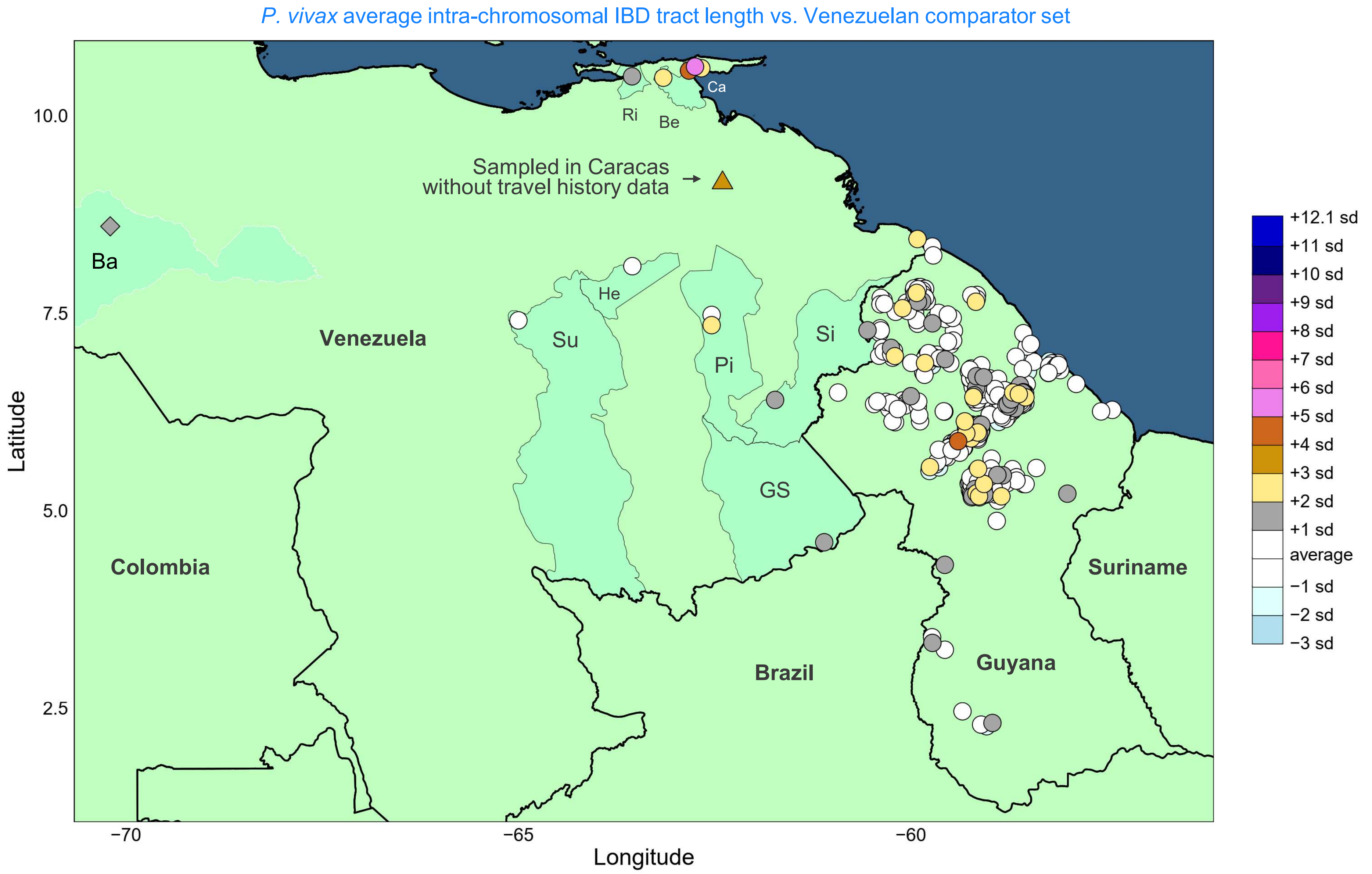

(Supp. Fig. 11 – continues on next page)

Supplementary Fig. 11 Differentiation between Guyanese and Venezuelan *P. falciparum* and *P. vivax* samples. Samples represent 2020-21 (Guyana) and 2015-16 + 2019 (Venezuela). **a)** IBD density curve for *P. falciparum* sample-pairs representing within-Guyana comparisons (G-G, solid line) and Guyana-Venezuela comparisons (G-V, dashed line). **b)** For *P. falciparum*, map of maximum genome-wide IBD vs. any Venezuelan sample (MaxVZ). **c)** For *P. falciparum*, map of genome-wide IBD vs. G4G410 (including comparison of G4G410 to itself (i.e., 100% IBD)). **d)** For *P. falciparum*, map of genome-wide IBD vs. G4G1043 (including comparison of G4G1043 to itself (i.e., 100% IBD)). **e)** IBD density curve for *P. vivax* sample-pairs representing within-Guyana comparisons (G-G, solid line) and Guyana-Venezuela comparisons (G-V, dashed line). **f)** For *P. vivax*, map of maximum genome-wide IBD vs. any Venezuelan sample (MaxVZ). **g)** Principal component analysis in *P. falciparum* (left) and *P. vivax* (right). Sample points plotted on PC1 vs. PC2 are colored according to epidemiological zone, and shape indicates country. All Venezuelan samples are classified to a single zone (yellow triangles). Analysis uses sites with >2% minor allele frequency and no missing genotype calls. **h)** For *P. falciparum*, map of average genome-wide IBD vs. any Venezuelan sample. **i)** For *P. falciparum*, map of maximum intra-chromosomal IBD tract length vs. any Venezuelan sample. **j)** For *P. falciparum*, map of average intra-chromosomal IBD tract length vs. any Venezuelan sample. **k)** For *P. vivax*, map of average genome-wide IBD vs. any Venezuelan sample. **l)** For *P. vivax*, map of maximum intra-chromosomal IBD tract length vs. any Venezuelan sample. **m)** For *P. vivax*, map of average intra-chromosomal IBD tract length vs. any Venezuelan sample. In each map, points are colored based on the standard deviation of the IBD metric from the mean (see color scale at right). Points with higher values are plotted above points with lower values and a slight jitter (±0.1 degree) is applied to all latitude and longitude coordinates to reduce overlap. Circles represent samples with travel history recorded to the locality level (i.e., using specific coordinates). Squares represent samples with travel history recorded to the municipality level (coordinates are placed within known malaria areas of the municipality (black perimeter)). Travel history for sample CEM526_Pv-2 (diamond) is recorded only to the state level (Barinas (Ba), white perimeter)). Its coordinates are placed arbitrarily within Ba. Samples CEM541_Pv-9, PW0065-C, and SPT26229 (triangles and/or text annotation) do not contain any travel history details. Star symbols are used for clonal samples with representation in both Guyana and Venezuela. Municipalities are abbreviated within the Venezuelan states of Bolívar (EC (El Callao), GS (Gran Sabana), He (Heres), Pi (Piar), Sifontes (Sifontes), Su (Sucre)), Sucre (Be (Benítez), Ca (Cajigal), and Ri (Ribero)), and Miranda (Gu (Guaicaipuro)).

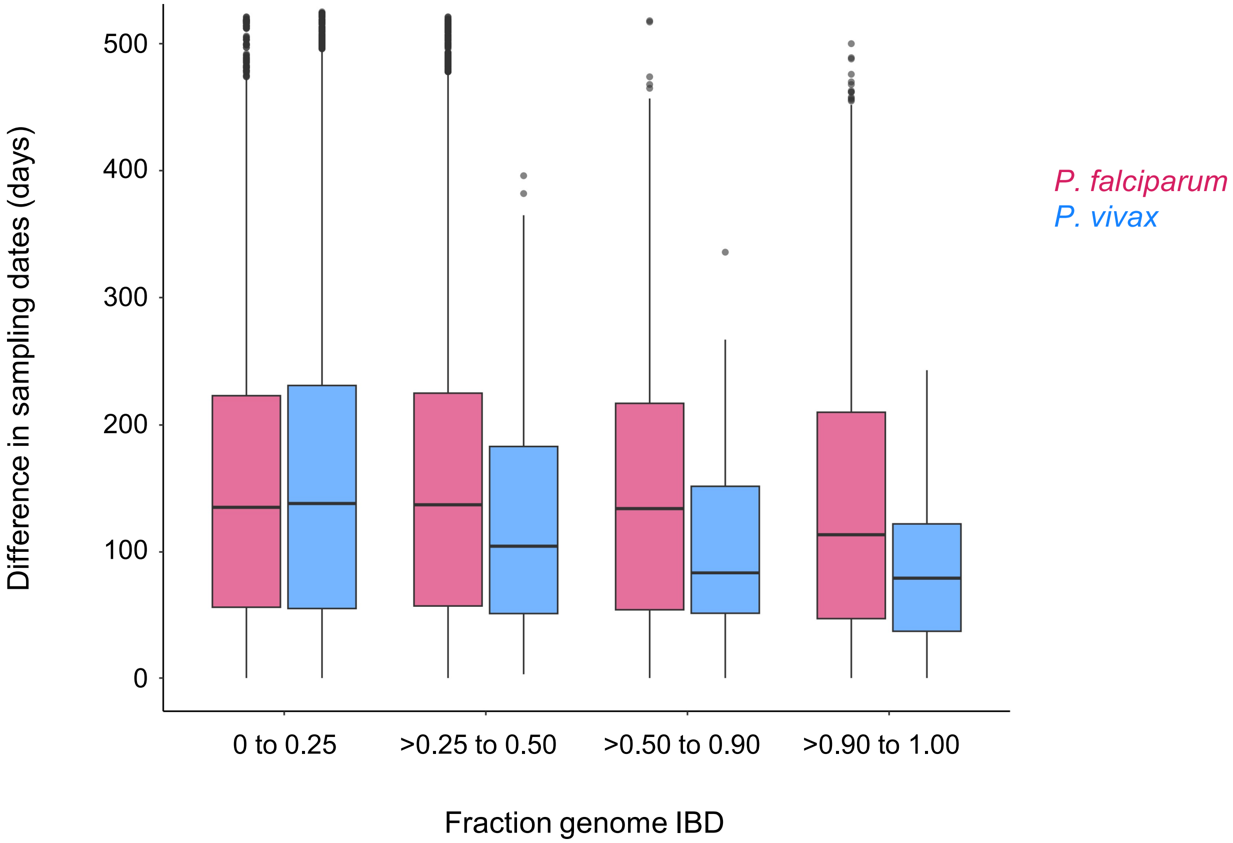

Supplementary Fig. 12 *P. falciparum* and *P. vivax* IBD with respect to time difference in sampling dates in Guyana. Boxplots summarize variation (median and quartiles) in time (days) between sampling for different IBD categories (increasing from low to high (clonal) on x-axis) in 2020-21.

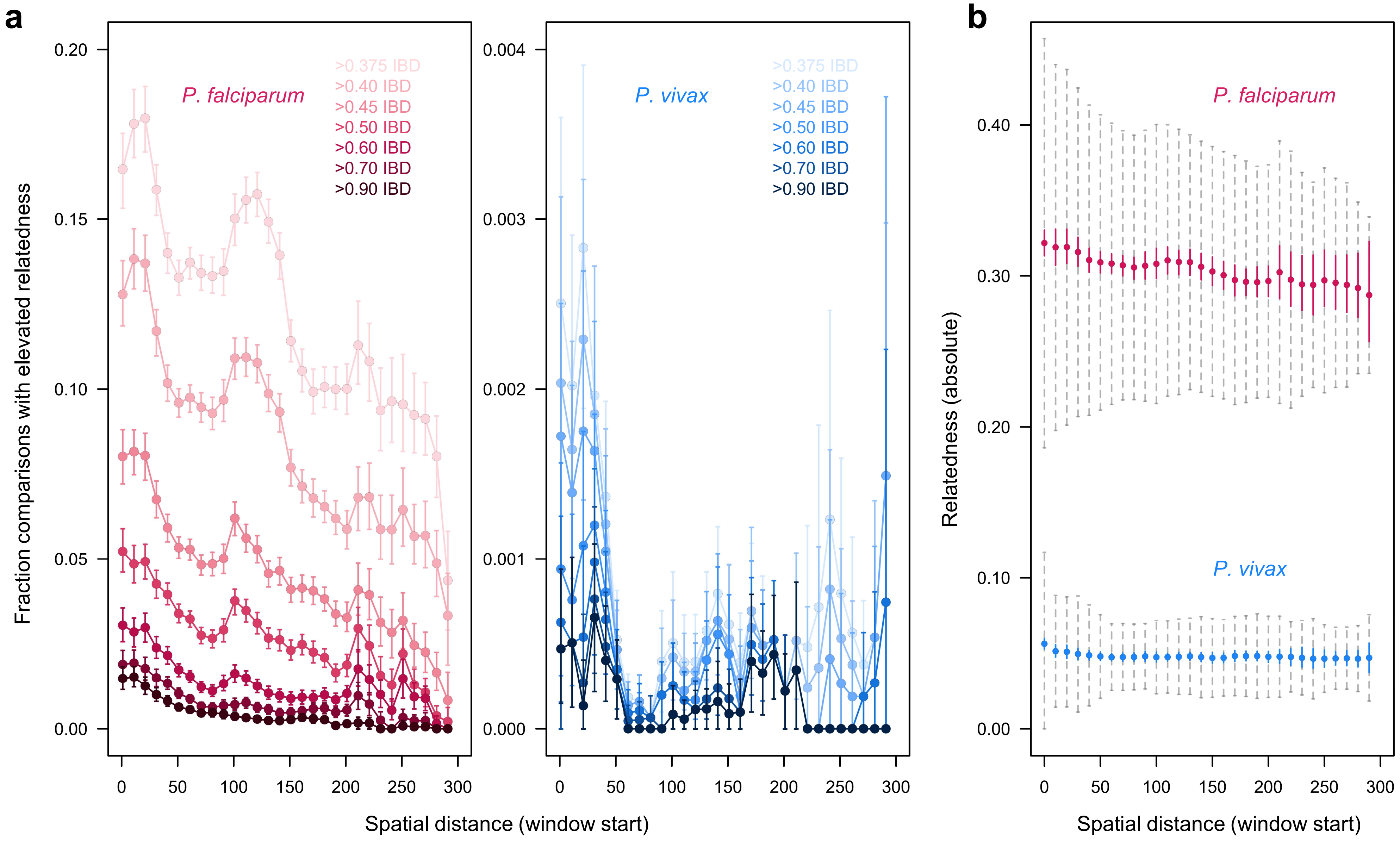

Supplementary Fig. 13 Use of different relatedness metrics for isolation-by-distance analysis in *P. falciparum* and *P. vivax* in Guyana. **a)** The spatial distance between inferred infection sites is plotted against the frequency of elevated parasite relatedness in 2020-21. Elevated relatedness is defined using various thresholds of pairwise IBD (colors). **b)** The y-axis shows absolute relatedness instead of the frequency of elevated relatedness. Points represent means and grey dashed lines represent ±1 standard deviation (sd). Methods for all three plots are otherwise equivalent to Fig. 3b (sliding window size = 30 km, step size = 10 km, 90% confidence intervals (solid colored bars) generated by bootstrapping 1000x, and clonal *P. falciparum* groups represented by ≥10 members excluded from analysis). Results indicate robust spatiogenetic patterns across methods but residuals rapidly increase for *P. falciparum* when lowering thresholds below 0.50 IBD or when interpreting IBD as a quantitative variable (e.g., mean and sd). This increased noise likely stems from high background relatedness in a historically inbred parasite population.

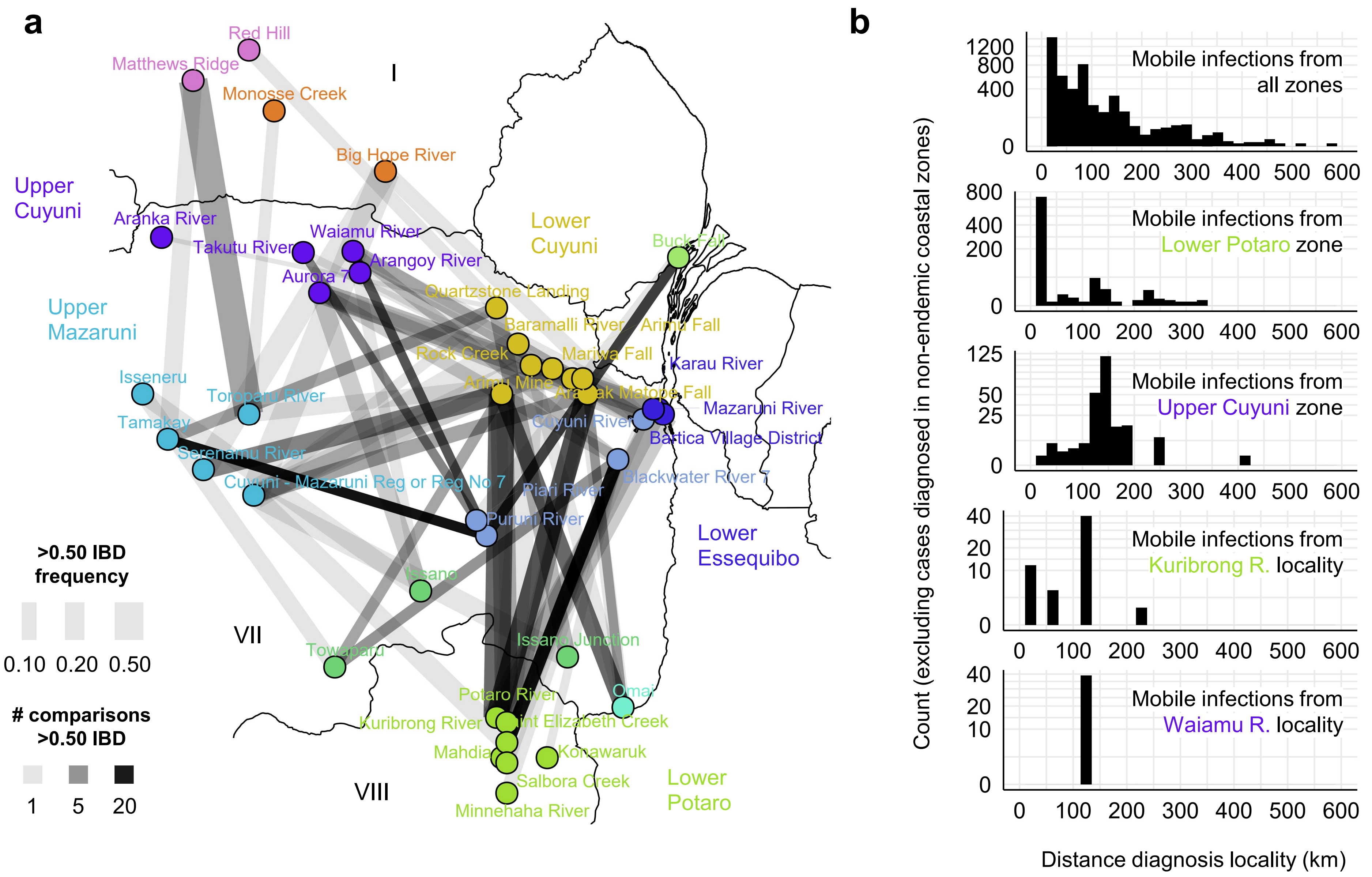

Supplementary Fig. 14 Geographic comparisons underlying a *P. falciparum* relatedness uptick at 111 - 132 km. a) Segments represent infection locality pairs for which non-zero >0.50 IBD frequency occurs. Segment widths and opacities represent the percent and total number of pairwise comparisons exhibiting >0.50 IBD. Localities are colored by epidemiological zone. **b)** Histograms show distances inferred between non-matching diagnosis and infection localities in the 2019 epidemiological database (excluding cases diagnosed in East/West of Georgetown (GT) and Greater GT zones). Distances often fall within the 111 - 132 km range when cases involve infection at prominent nodes of parasite genetic connectivity observed in the map. Movement to Bartica underlies most of the 111 - 132 km distance signal found for infection from Lower Potaro (98%), Upper Cuyuni (94%), Kuribrong River (100%), and Waiamu River (100%). Infection localities are inferred based on reported patient stay two weeks prior to diagnosis.

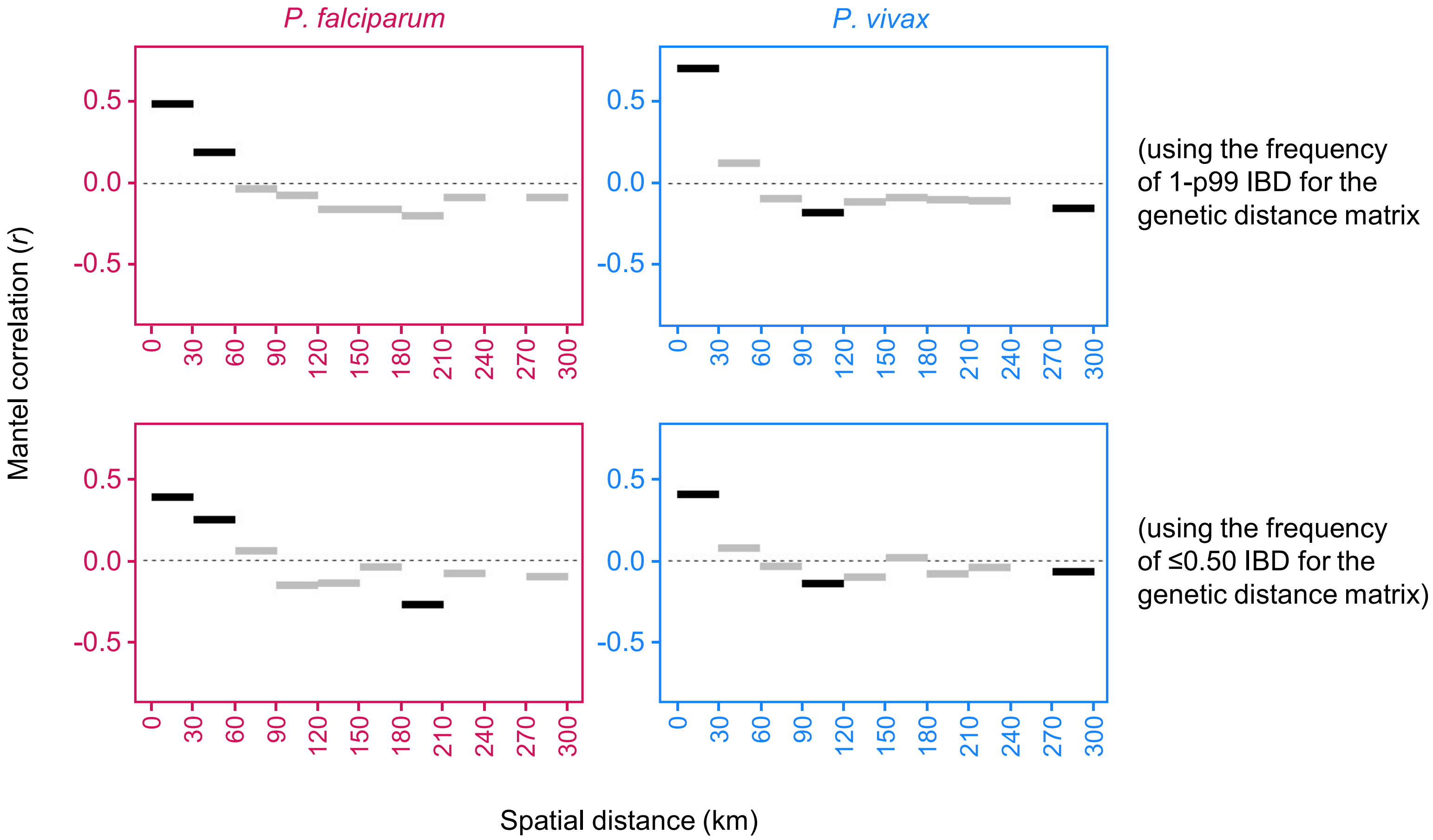

Supplementary Fig. 15 Mantel correlograms for *P. falciparum* and *P. vivax* in Guyana. Mantel correlation coefficients (r) are plotted on the y-axis for successive (non-overlapping) spatial distance classes (bars) separating infection localities in 2020-21. Black bars indicate significant r ($p < 0.05$) between genetic and spatial distance matrices. Top plots use 1-p99 IBD frequency and bottom plots use ≤0.50 IBD frequency for the genetic distance matrix. Positive *r* represents isolation-by-distance, i.e., that genetic distance increases with spatial distance.

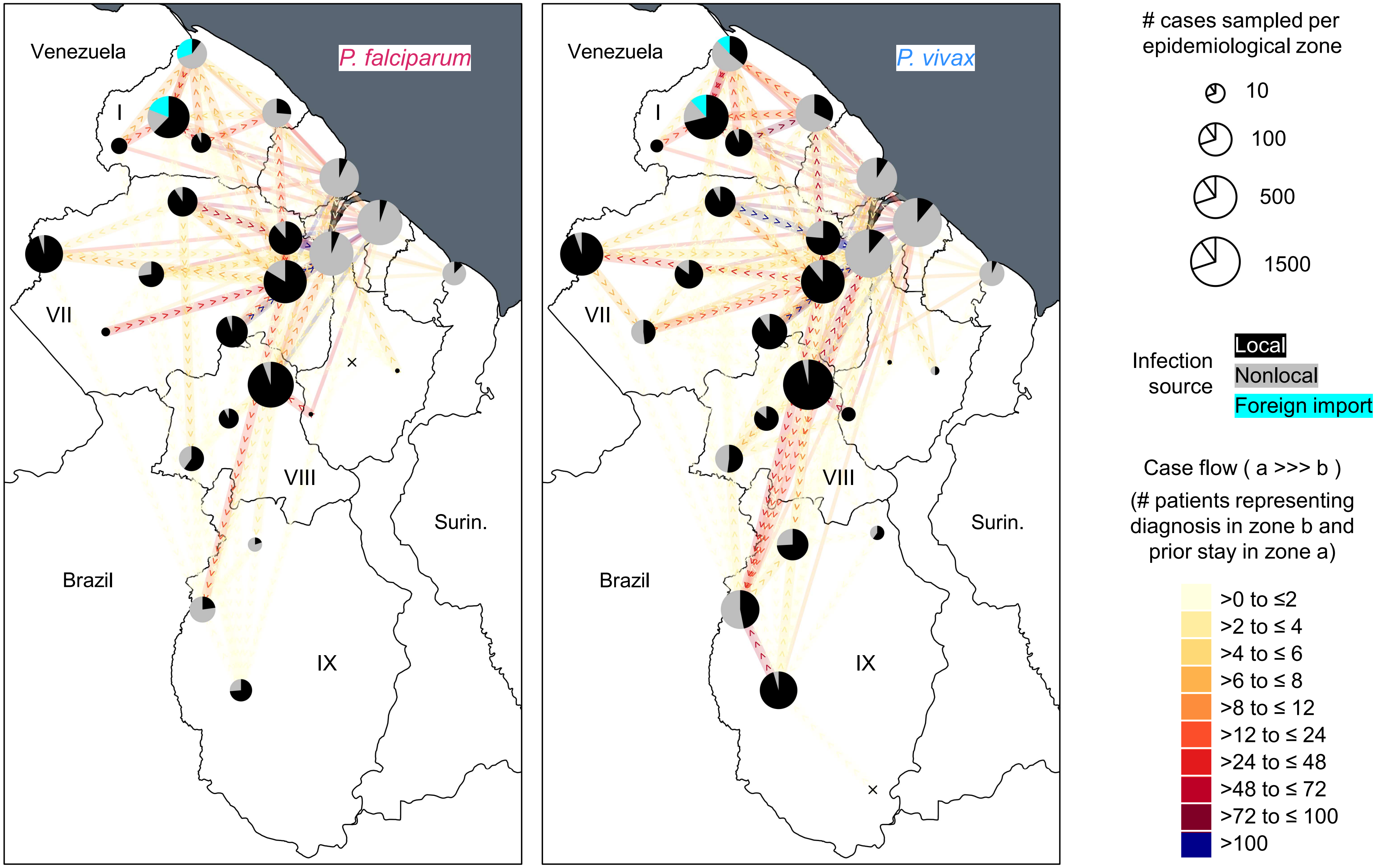

Supplementary Fig. 16 *P. falciparum* and *P. vivax* case flow in Guyana. Pie icon sizes represent the number of cases sampled (diagnosed) within each epidemiological zone in 2019. Black slices represent the fraction of cases representing patients that reported having stayed in the same epidemiological zone 2 weeks prior to diagnosis. Grey slices represent the fraction of cases representing patients that reported having stayed in a different epidemiological zone 2 weeks prior to diagnosis. Case flow segments are mapped for such 'non-local' cases. These segments connect the reported location of prior stay to the location of diagnosis. Arrows are used if the location of diagnosis is considered an endemic zone (e.g., for this reason only solid lines connect to coastal zones around Georgetown). The number of case flow events recorded between zones is represented by segment color, increasing from yellow to red and blue (see scale). Zones in which sampling did not occur but which featured as locations of prior stay are indicated by cross symbols. The analysis uses 13,641 cases for which information on location of diagnosis and on location 2 weeks prior to diagnosis is available.

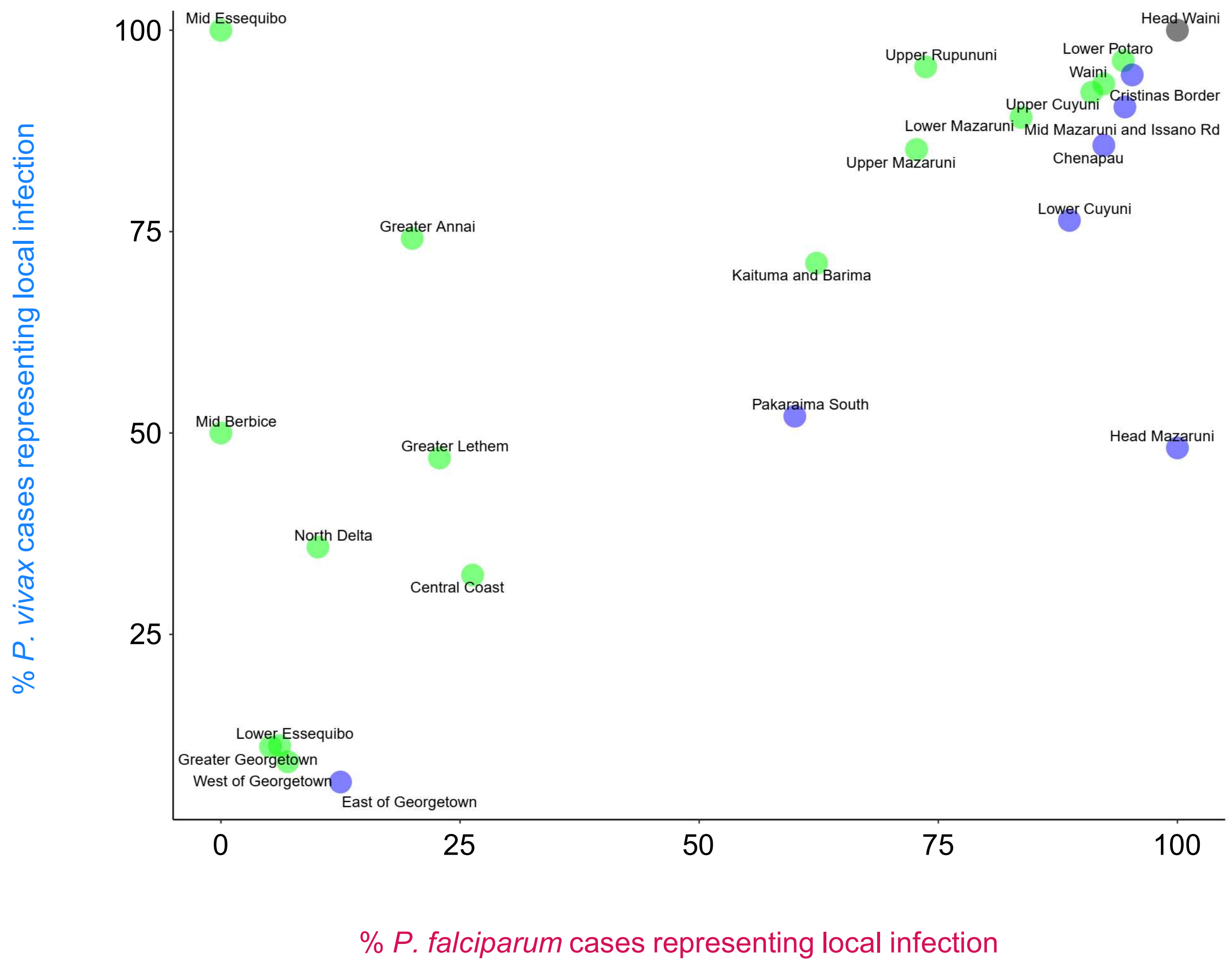

Supplementary Fig. 17 Rates of local infection for *P. falciparum* and *P. vivax* in Guyana. For each epidemiological zone (points), the percentage of *P. falciparum* cases representing local infection (x-axis) is plotted against the percentage of *P. vivax* cases representing local infection (y-axis) in 2019. Values correspond to black pie slices in Supplementary Fig. 16. Blue is used when higher values occur for *P. falciparum*, green is used when higher values occur for *P. vivax*, and black is used when values are equal between species.

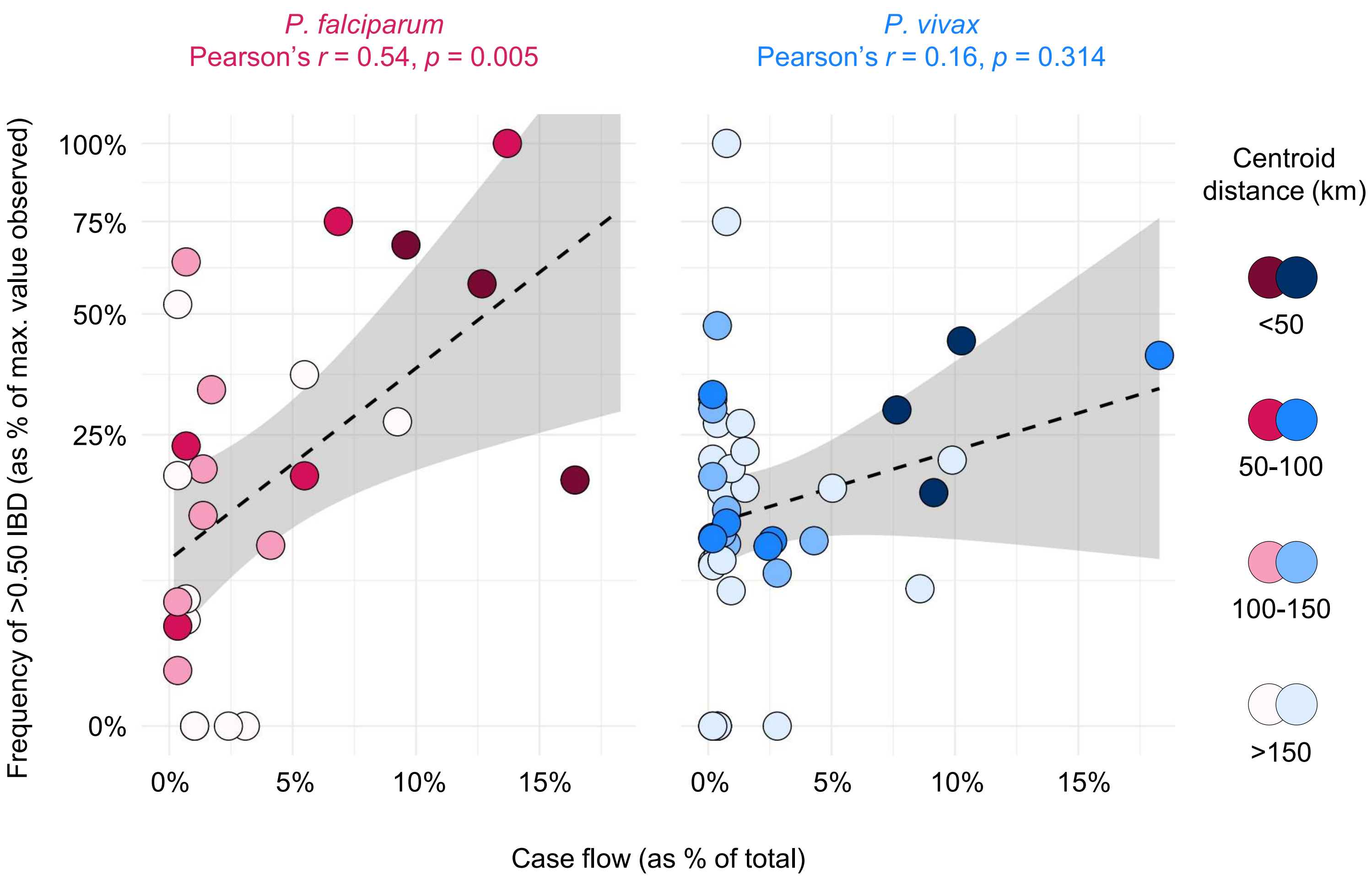

Supplementary Fig. 18 Relationship between the relative frequency of parasite p99 IBD and patient case flow detected between epidemiological zones in *P. falciparum* and *P. vivax* in Guyana. Genetic data (y-axis) represents 2020-21. Epidemiological (case flow) data (x-axis) represents 2019. Point color indicates the spatial distance separating the zones being compared. Comparisons represented by ≤50 comparisons are excluded. Pearson correlation is statistically significant in *P. falciparum* but not in *P. vivax*. Grey shading indicates 95% confidence intervals predicted by linear regression (dashed line).

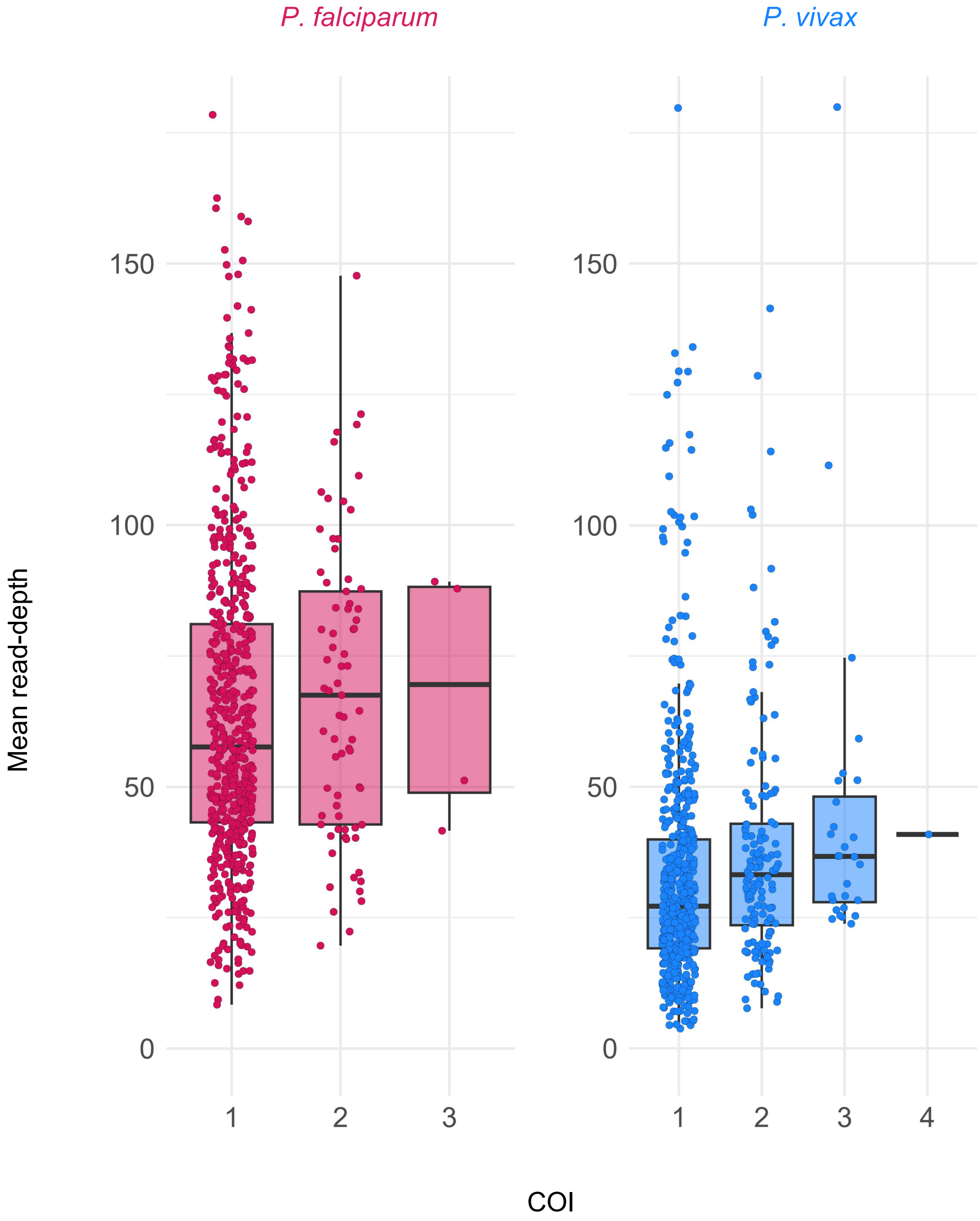

Supplementary Fig. 19 Relationship between complexity of infection (COI) values and sequencing depth in *P. falciparum* and *P. vivax* samples. Boxplots summarize variation (median and quartiles) in mean read-depth associated with each sample (points). Samples are grouped by COI level (1 - 4) along the x-axis. One outlier *P. falciparum* sample (G7B667, mean read-depth = 262.9) is not shown. Mean read-depth differs significantly between species (Welch's t-test, $t = 20.324$, $p < 0.001$) and for monoclonal vs. polyclonal samples in *P. vivax* (Welch's t-test, $t = 2.777$, $p = 0.006$), but not in *P. falciparum*. Mean read-depths do not differ by COI level within either species (Tukey's HSD test).

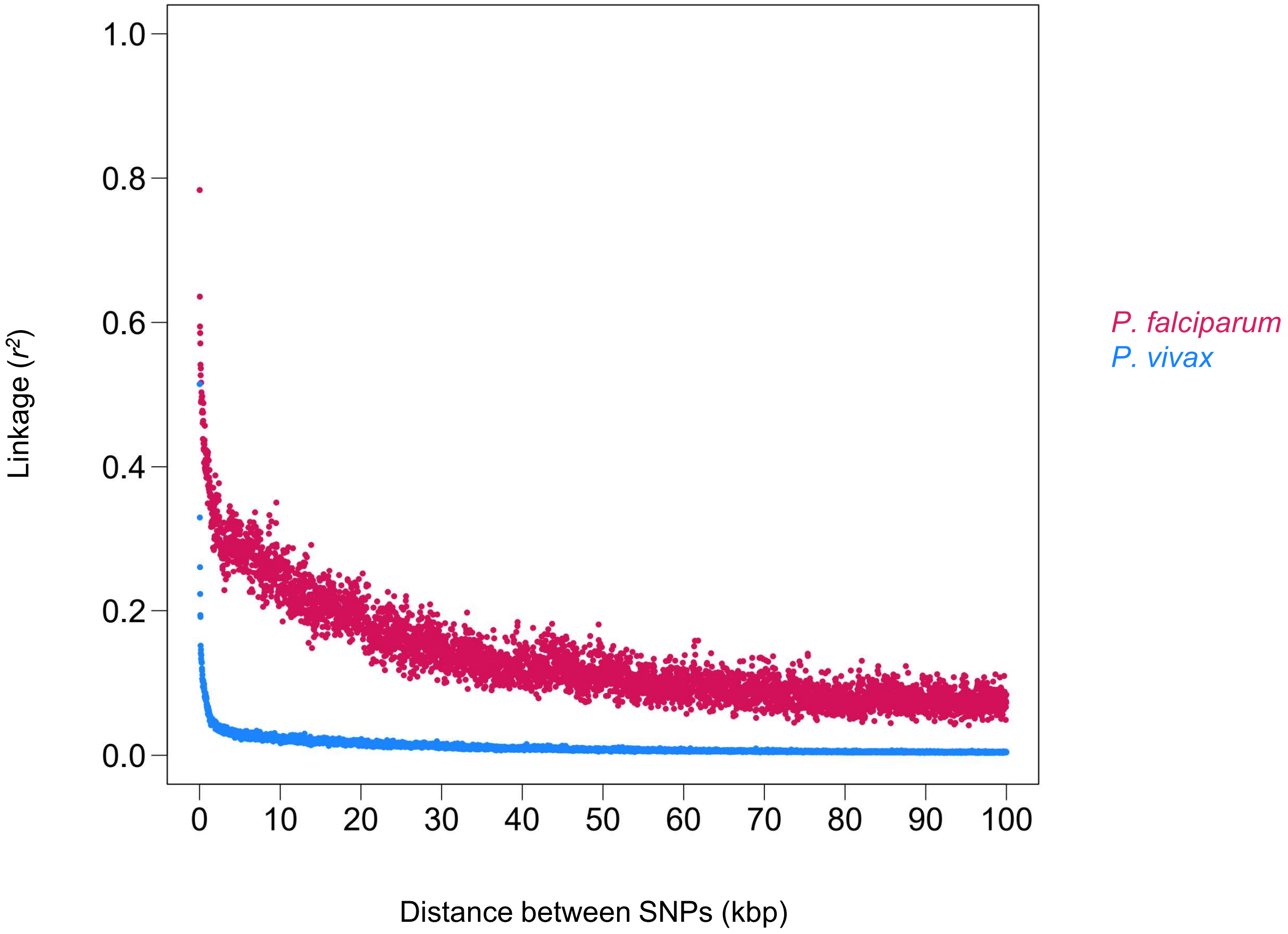

Supplementary Fig. 20 Linkage decay in *P. falciparum* and *P. vivax* in Guyana. Average linkage values (r^2) between SNP sites are plotted in sliding 10 kbp windows (step size = 200 bp) for samples from 2020-21.

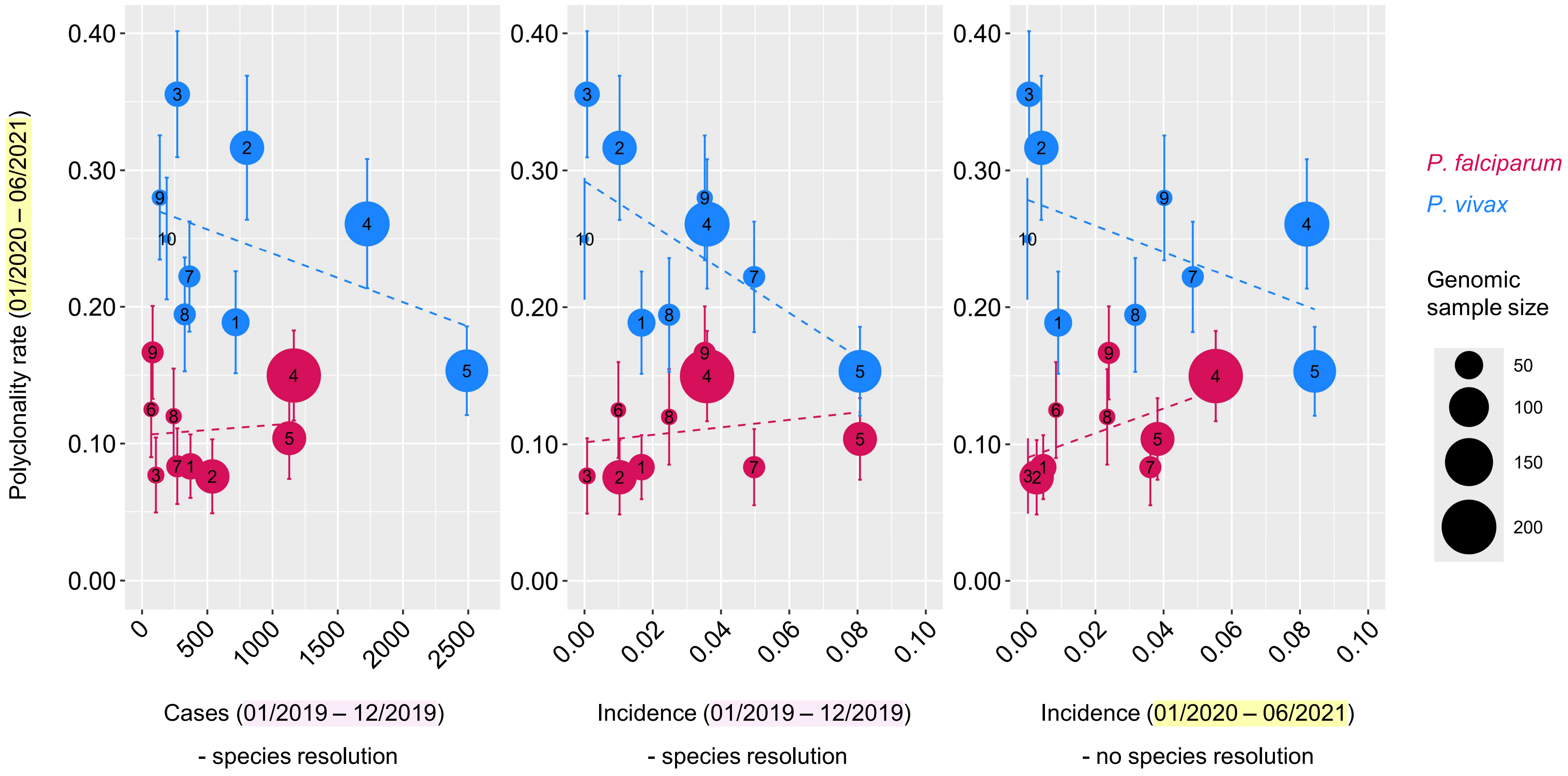

Supplementary Fig. 21 Polyclonality rates vs. absolute case counts and incidence estimates for *P. falciparum* and *P. vivax* in Guyana. Points represent epidemiological zones. Only zones with at least 20 genomic samples (see size key) are included in analysis (Kaituma and Barima (1), Lower Cuyuni (2), Lower Essequibo (3), Lower Mazaruni (4), Lower Potaro (5), Mid Essequibo (6), Mid Mazaruni and Issano Rd (7), Upper Cuyuni (8), Upper Mazaruni (9), Greater Georgetown (10)). The left plot compares 2019 case counts of each species (x-axis) to polyclonality rates representing 2020-21 (y-axis). The other two plots instead use incidence (absolute counts adjusted by population density) on the x-axis. In the center plot, incidence is estimated by dividing the average monthly 2019 case count of each species by the LandScan population size projection (see Methods) within a 45 km radius of the zone's centroid coordinate. Interpretation of the left and center plots is however complicated by temporal mismatch between x and y axes. We therefore acquired an additional case count list for 01/2020 – 06/2021 from the Guyana Ministry of Health to compute the right plot. This list however only contains total malaria counts (no species-level resolution). The linear regressions (dashed lines) are not statistically significant in any of the three plots. Error bars represent 95% confidence intervals from bootstrapping 100x.

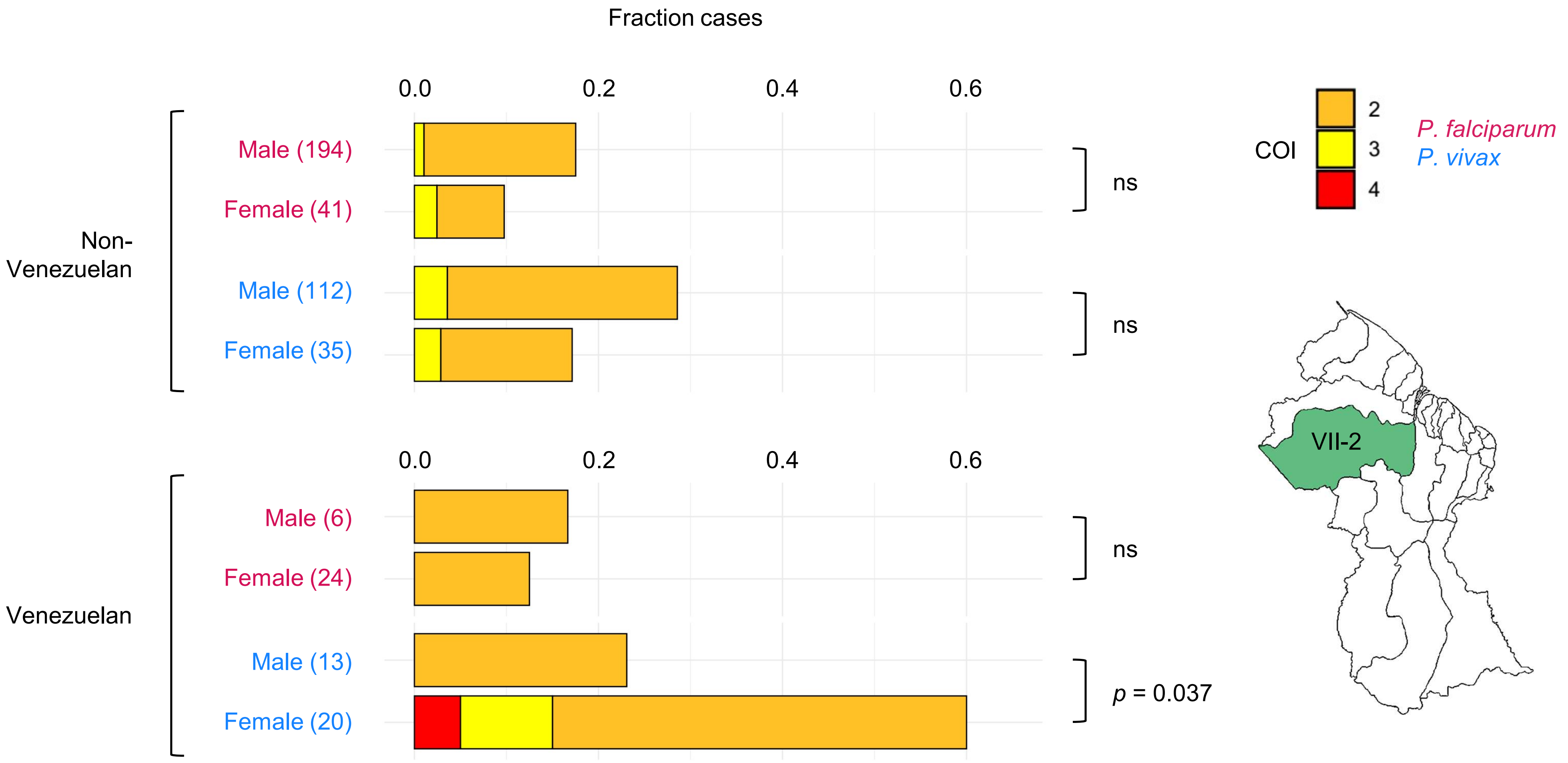

Supplementary Fig. 22 Complexity of infection values for *P. falciparum* and *P. vivax* in Venezuelan and non-Venezuelan patients with infections attributed to NDC VII-2 in Guyana. NDC VII-2 is highlighted in green in the map. Upper plots represent patients of non-Venezuelan nationality and lower plots represent patients of Venezuelan nationality (see large brackets at left). For each malaria species and patient gender, horizontally stacked bars represent complexity of infection (COI) values contributing to total observed polyclonality rate (full length of bar on x-axis) in 2020-21. Orange = 2 strains, yellow = 3 strains, and red = 4 strains. *P. vivax* polyclonality rate is significantly elevated in female Venezuelan patients vs. male Venezuelan patients (Chi-squared test). Differences are non-significant (ns) for other indicated comparisons (see small brackets at right).

VECTOR CONTROL SERVICES - MINISTRY OF PUBLIC HEALTH

M1 (Revised Dec, 2016) **Malaria Daily Case Register (Health Facilities)** Epidemiological week

SAMPLE DATA

**Sample Number:**	**Locality:**	**Health Facility:**

**Date taken**	**Region**	***Case***		***Detection method***			**Name** (who took sample)
					**Active**		
dd/mm/yyyy		**New**	**Recheck**	**Passive**	**Mass Blood Survey**	**Fever Case Survey**	

PATIENT DATA

Age, yrs | Month: (if < 1 yr) | **Sex:** M \| F | **Pregnant** N \| Y | **If pregnancy (# Months)**

Current Address | **Locality** | **Region** | **Nationality**

Ethnic Group (for Guyanese): African | Amerindian | East Indian | Chinese | Portuguese | European | Mixed

Where patient stayed 2 weeks ago: Locality | Region | Country

Symptoms started on: dd / mm / yyyy

Fever: **Current | Recent | No fever** — OTHER SYMPTOM(S)

LABORATORY TESTS PERFORMED

RDT: Yes | No — **Date of RDT:** dd / mm / yyyy — **RDT Result: Neg (-) | Pos (+)** — ***P. vivax/malariae*** | ***P. falciparum*** | **Mixed**

Slide

**Date of slide**	**Neg**	**Pos**	*P. vivax*		*P. falciparum*		*P. malariae*		Mixed	
dd/mm/yyyy	-	+	***Plasmodium vivax* Density**			***P. malariae* Density**			***P. falciparum* Density**	
			Rings I Gametes I WDC			Rings I Gametes I WDC			Rings I Gametes I WDC	

Examination Date: dd / mm / yyyy — **Microscopist Name:**

Treatment started on: dd / mm / yyyy — **# of CoArt** — **# CQ 150mg** — **# PQ 15 mg** — **# PQ 7.5 mg**

CoArt= Coartem; **CQ**= Chloroquine; **WBC**= White blood cells; **PQ**= Primaquine — **ALL fields should be filled.**

O**riginal** (Regional Health Office), **Copy** (Health Facility); **Fever: Current** (0 - 5 days), **Recent** (6 - 30 days)**, No fever** (in the last 31 days)**.**

Supplementary Fig. 23 Malaria case form underlying patient metadata records analyzed from Guyana. Form records of sampling date, sampling location, patient gender, patient age, patient nationality, patient ethnicity, location of patient stay 2 weeks prior to diagnosis, and infecting species (microscopy or RDT result) were provided to study authors using anonymized patient codes. Only cases marked ‘New’ and ‘Passive’ were analyzed. The same form was used for 2019 and 2020-21 sample sets.

Supplementary Table 1 Malaria case composition in Guyana (2019).

**Composition** (by microscopy and/or rapid diagnostic analysis)	**Cases**
P. vivax	11938
P. falciparum	5727
P. falciparum + *P. vivax*	993
P. malariae + *P. vivax*	91
P. malariae	39
P. falciparum + *P. malariae* + *P. vivax*	8
Plasmodium sp. gametocytes + *P. vivax*	6
Plasmodium sp. gametocytes	12
P. falciparum + *P. malariae*	6
Plasmodium sp. gametocytes + *P. malariae* + *P. vivax*	2

Supplementary Text 1

Relationships between samples collected in Guyana and Venezuela

We assessed whether our small comparator set from Venezuela (22 monoclonal samples) could indicate the presence of cross-border parasite genetic divergence and transmission of imported strains within Guyana. The Venezuelan comparator set primarily represents infections from the eastern states of Bolívar and Sucre. Two *P. falciparum* samples (PW0065-C and SPT26229) and one *P. vivax* sample (CEM541_Pv-9) lack travel history data. In *P. falciparum*, the IBD distribution for sample-pairs representing comparisons within Guyana (median = 0.283) appeared right-shifted (Wilcoxon test, $W = 57691375$, $p < 0.001$) relative to the IBD distribution for sample-pairs representing cross-border comparisons (median = 0.261, Supplementary Fig. 11a). To further visualize this divergence signal in *P. falciparum* and to screen for imported transmission, we mapped the highest IBD value observed for each sample with respect to samples representing Venezuela ('MaxVZ', Supplementary Fig. 11b). Only three infections from Guyana showed aberrant (>2 sd above average) maxVZ. Two of these three (G4G410 and G4G1043) were clonal (>0.90 IBD) with respect to Venezuelan infections Venez_001_F1 and PW0065-C (Supplementary Fig. 11c,d). Highly aberrant (>3 sd above average) IBD relative to G4G410 or G4G1043 occurred in five of nine Venezuelan infections but in just one of 527 Guyanese infections. These observations suggest that G4G410 and G4G1043 represent cases of imported transmission from Venezuela into Guyana. Observing just two such cases in the sample set suggests that imported transmission is infrequent (despite health posts near the border frequently experiencing foreign cases – see cyan pie slices in Supplementary Fig. 16) but verification is required with larger Venezuelan sample sets.

In *P. vivax,* IBD distributions for Guyana-Guyana and Guyana-Venezuela comparisons were also statistically distinguishable ($p < 0.001$) but were very close in absolute overlap (median 0.046 vs. 0.041 (respectively), Supplementary Fig. 11e). Although scarce, aberrant maxVZ values (>2 sd above average) occurred within Guyana more frequently in *P. vivax* (7/474) than in *P. falciparum* (3/528) and occurred within Venezuela less frequently in *P. vivax* (5/13) than in *P. falciparum* (6/9) (Supplementary Fig. 11f vs. Supplementary Fig. 11b).

Principal component analysis using direct SNP data (IBS instead of IBD, Supplementary Fig. 11g) did not help clarify Guyana-Venezuela population patterns in either species. Maps of alternative IBD features (e.g., maximum intra-chromosomal IBD tract length) were also difficult to interpret (Supplementary Fig. 11h-m).

Supplementary Text 2

Spatial comparisons underlying elevated P. falciparum relatedness at 100 - 130 km

To examine the spatial comparisons underlying a prominent uptick in *P. falciparum* >0.50 IBD frequency observed at x-axis position 100 (i.e., distance window 100 - 130 km) in Fig. 3b, we first repeated sliding window analyses using smaller window sizes and steps. This allowed us to pinpoint the uptick range more specifically as 111 - 132 km. Next, we mapped segments representing all site pairs belonging to the 111 - 132 km distance class and featuring between-site sample comparisons with >0.50 IBD (Supplementary Fig. 14a). The map shows several segments converging into the Lower Essequibo (LE) zone, especially connecting the Lower Potaro (LP) zone (e.g., Kuribrong River) and Bartica (LE) and connecting the Upper Cuyuni (UC) zone (e.g., Waiamu River) and Bartica (LE). Several segments connecting LP and the Lower Cuyuni (LC) zone (e.g., Kuribrong River vs. Oko River) and fanning out from Puruni River in the Lower Mazaruni (LM) zone are also evident.

This uneven signal of elevated parasite relatedness among a limited set of nodes may reflect elevated co-visitation by infected hosts. We assessed whether the 2019 epidemiological database supports this hypothesis by assessing distances between the localities at which patients were diagnosed and the localities at which patients reported having stayed 2 weeks prior to diagnosis (used as an estimate of the geographic source of infection throughout this study). We focused only on cases for which infection and diagnosis localities do not match (i.e., cases in which host travel away from the infection source appears to have occurred). Our analysis also excluded ‘GT’ cases, i.e., cases diagnosed in West of Georgetown, Greater Georgetown, and East of Georgetown zones (considered non-endemic and thus less likely to contribute to onward parasite transmission/gene flow).

While the resultant distance distribution is clearly left-skewed (i.e., long distance movements appear less common than short distance movements) and shows no aberrations at 111 - 132 km (Supplementary Fig. 14b, top panel), this pattern changes when we further subset analysis to cases for which prior stay

represents LP or UC (Supplementary Fig. 14b, second and third panels). We now see clear travel distance modes within the range of 111 - 132 km. This is also true when subsetting to cases which indicate prior stay specifically at Kuribrong River or Waiamu River (Supplementary Fig. 14b, bottom two panels), two key nodes connecting LP and UC to LC and LE (Supplementary Fig. 14a). The vast majority of the cases representing this distance range were diagnosed in Bartica Village District (details in Supplementary Fig. 14 legend). Movement patterns from the 2019 epidemiological database therefore offer some support to the hypothesis that elevated co-visitation, specifically involving LP - LE and UC - LE, contribute to elevated >0.50 IBD frequency in the associated distance range.

The epidemiological database does not, however, mirror >0.50 IBD segment patterns representing LP - LC comparisons or those involving Puruni River (LM). In fact, the database contains zero cases of host movement from LP to LC or from LC to LP and only 4 of 665 non-GT cases which involve movement to or from Puruni River represent the 111 - 132 km distance range.

Incongruences between parasite IBD-based connectivity signals and the patient travel history information may reflect parasite relatedness patterns unrelated to contemporary gene flow (e.g., historic clonal expansions). The limited resolution at which patient movement could be characterized using the epidemiological database likely also plays a role. Given just two recorded localities per patient, details on transit routes and stop-over sites intervening infection and diagnosis events are hidden from view. It is very possible, for example, that many cases representing infection in LP and diagnosis in Bartica involve patients which did not initiate travel to Bartica or associated areas for the purpose of medical attention. Mahdia, the capital of Region VIII, is a much easier option within LP when medical attention is the goal. Bartica is relatively easily accessible from LP (e.g., <4 hours by land according to the Ministry of Infrastructure), and represents the primary logistics and departure hub for mining activities along the Mazaruni and Cuyuni Rivers. Examples of relatively accessible mining areas include Oko River and Arimu Mine (see nodes in Supplementary Fig. 14b). An infected host from LP working in such areas accessed in Bartica is very likely to return to Bartica for medical attention once symptoms arise.

Supplementary References

1. Tyers, M. riverdist: river network distance computation and applications. R package version 0.16.3 (2024).

2. Weiss, D. J. et al. A global map of travel time to cities to assess inequalities in accessibility in 2015. Nature 553, 333–336 (2018).

3. van Etten, J., de Sousa, K. & Marx, A. gdistance: distances and routes on geographical grids. R package version 1.6.4 (2023).
