# Supplementary material for: Contrasting genomic epidemiology between sympatric Plasmodium falciparum and Plasmodium vivax populations
Source: Nat Commun. 2024 Sep 30;15:8450. doi: 10.1038/s41467-024-52545-6 (PMC11442626; doi:10.1038/s41467-024-52545-6)
Supplement: Supplementary file 3 — Description of Additional Supplementary Files [file 41467_2024_52545_MOESM3_ESM.pdf]

Description of Additional Supplementary Files

File Name: Supplementary Data 1
Description: **Metadata for the genomic sample set.** Locations represent inferred sources of infection based on reported patient stay 2 weeks prior to diagnosis.
